# HNF1A binds and regulates the expression of SLC51B to facilitate the uptake of estrone sulfate in human renal proximal tubule epithelial cells

**DOI:** 10.1038/s41419-023-05827-8

**Published:** 2023-05-03

**Authors:** Jun Wei Chan, Claire Wen Ying Neo, Soumita Ghosh, Hyungwon Choi, Su Chi Lim, E. Shyong Tai, Adrian Kee Keong Teo

**Affiliations:** 1 Stem Cells and Diabetes Laboratory, Institute of Molecular and Cell Biology (IMCB), Agency for Science, Technology and Research (A*STAR), Singapore, 138673 Singapore; 2 Department of Medicine, Yong Loo Lin School of Medicine, National University of Singapore, Singapore, 119228 Singapore; 3 Khoo Teck Puat Hospital, Singapore, 768828 Singapore; 4 Saw Swee Hock School of Public Health, National University of Singapore, Singapore, 117549 Singapore; 5 Lee Kong Chian School of Medicine, Nanyang Technological University, Singapore, 308232 Singapore; 6 Precision Medicine Translational Research Programme, Yong Loo Lin School of Medicine, National University of Singapore, Singapore, 119228 Singapore; 7 Department of Biochemistry, Yong Loo Lin School of Medicine, National University of Singapore, Singapore, 117596 Singapore

**Keywords:** Disease model, Differentiation

## Abstract

Renal defects in maturity onset diabetes of the young 3 (MODY3) patients and *Hnf1a*^-/-^ mice suggest an involvement of HNF1A in kidney development and/or its function. Although numerous studies have leveraged on *Hnf1α*^*-/-*^ mice to infer some transcriptional targets and function of HNF1A in mouse kidneys, species-specific differences obviate a straightforward extrapolation of findings to the human kidney. Additionally, genome-wide targets of HNF1A in human kidney cells have yet to be identified. Here, we leveraged on human in vitro kidney cell models to characterize the expression profile of HNF1A during renal differentiation and in adult kidney cells. We found *HNF1A* to be increasingly expressed during renal differentiation, with peak expression on day 28 in the proximal tubule cells. HNF1A ChIP-Sequencing (ChIP-Seq) performed on human pluripotent stem cell (hPSC)-derived kidney organoids identified its genome-wide putative targets. Together with a qPCR screen, we found HNF1A to activate the expression of *SLC51B*, *CD24*, and *RNF186* genes. Importantly, *HNF1A*-depleted human renal proximal tubule epithelial cells (RPTECs) and MODY3 human induced pluripotent stem cell (hiPSC)-derived kidney organoids expressed lower levels of *SLC51B*. SLC51B-mediated estrone sulfate (E1S) uptake in proximal tubule cells was abrogated in these HNF1A-deficient cells. MODY3 patients also exhibit significantly higher excretion of urinary E1S. Overall, we report that SLC51B is a target of HNF1A responsible for E1S uptake in human proximal tubule cells. As E1S serves as the main storage form of nephroprotective estradiol in the human body, lowered E1S uptake and increased E1S excretion may reduce the availability of nephroprotective estradiol in the kidneys, contributing to the development of renal disease in MODY3 patients.

## Introduction

The hepatocyte nuclear factor 1 A (HNF1A) is a transcriptional activator predominantly expressed in the pancreas, liver and kidneys [1, 2], with cell type-specific functions. In the pancreas, HNF1A modulates insulin secretion in the pancreatic β-cells [3]. Heterozygous *HNF1A* mutation are known to cause maturity onset diabetes of the young 3 (MODY3) [4, 5]. Within the liver, *Hnf1a* orchestrates bile acid and plasma cholesterol metabolism in hepatocytes [6], as evidenced by the onset of hypercholesterolemia in *Hnf1a*^-/-^ mice [7]. In the kidney, loss of *Hnf1a* impairs renal proximal tubule reabsorption, leading to renal Fanconi syndrome [7, 8]. Together, these studies indicate that Hnf1a plays important and distinct physiological functions in the pancreas, liver and kidney.

To date, there have been efforts to uncover the transcriptional targets of HNF1A, albeit mainly in the pancreas and the liver [5, 9–14]. In pancreatic β-cells, HNF1A regulates the expression of genes involved in glucose metabolism and transport [9, 14] and the human insulin gene [10]. In the liver, HNF1A regulates the expression of glucose-6-phosphatase, which is important for gluconeogenesis [11]. Two studies have since used chromatin immunoprecipitation (ChIP) coupled with microarray (ChIP-on-chip) assays to unveil the transcriptional regulatory network downstream of HNF1A in the pancreas and liver based on HNF1A-bound targets [12, 13]. However, the genome-wide transcriptional targets of HNF1A in human kidney cells remain poorly understood.

Existing studies pertaining to *Hnf1a* in the kidney relied heavily on the use of rodent models. *Hnf1α*^*-/-*^ mice exhibit reduced expression of several renal transporter genes including the low affinity/high capacity glucose cotransporter *Sglt2* [15], the endocytic receptors *Lrp2* and *Cubn* [8], and the organic anion transporters *Oat1*/*Oat2/Oat3* [16]. Notably, many of these HNF1A target genes are expressed in the proximal tubule cells, congruent with the decline in proximal tubule function observed in *Hnf1a*^*-/-*^ mice [7, 8].

While there are relatively fewer studies documenting the role of HNF1A in the human kidney, MODY3 patients are reported to have a higher incidence of developing diabetic nephropathy [17, 18]. Furthermore, similar to the *Hnf1a*^-/-^ rodents, MODY3 patients tend to exhibit reduced renal threshold for glucose [15, 19]. However, unlike MODY3 patients who have a heterozygous *HNF1A* gene mutation, *Hnf1a* heterozygous (*Hnf1a*^+/-^) knockout mice do not exhibit renal defects [20]. To this end, it is widely recognized that species-specific differences between mice and humans can limit the applicability of pre-clinical studies performed on rodent models to the human setting [21]. Hence, this underscores the need to study the role and transcriptional targets of HNF1A in human kidney cell models.

Here, we used human pluripotent stem cell (hPSC)-derived kidney organoids, human primary renal proximal tubule epithelial cells (RPTECs) and human embryonic kidney 293 (HEK293AD) cells to investigate the direct transcriptional targets of HNF1A in human kidney cells. We examined the expression profile of *HNF1A* during the renal differentiation of hPSCs and demonstrated that HNF1A is expressed mainly in the proximal tubule cells. By performing ChIP-Seq on the hPSC-derived kidney organoids, we report the first effort at mapping out the genome-wide occupancy of HNF1A protein in human kidney cells. A targeted qRT-PCR screen was used to identify several novel direct transcriptional targets of HNF1A that are expressed in the proximal tubule cells. Amongst these, *SLC51B*, a member of the human solute carrier family known to mediate the transportation of estrone sulfate (E1S; the main storage form of estradiol in the human body [22]) across the basolateral membrane of proximal tubule epithelial cells, was selected for further study. Importantly, RPTECs depleted of HNF1A and MODY3 hiPSC-derived kidney organoids exhibit diminished ability to take up E1S, suggesting a dwindling pool of available estradiol in MODY3 patients harboring loss-of-function *HNF1A* gene mutations. Consistent with this, we found that excretion of urinary E1S was significantly elevated in MODY3 patients harboring *HNF1A* loss-of-function mutations. Decreased nephroprotective estradiol [23–25] may partly account for the heightened predisposition of MODY3 patients to develop renal complications. Altogether, our study identified *SLC51B* as a novel target of HNF1A in human kidney cells. Loss of HNF1A results in reduced SLC51B expression and subsequently E1S uptake in renal proximal tubule cells, that may lead to decreased renal protection. Modulation of E1S uptake in human kidney cells may be explored to mitigate *HNF1A* loss-of-function effects that can possibly contribute to renal deficiencies.

## Methods

### Cell culture

The use of donor-derived hiPSCs is approved under A*STAR IRB 2020-096. Informed consent has been obtained. hPSCs were cultured in TeSR^TM^-E8^TM^ or mTeSR^TM^1 (STEMCELL Technologies). RPTEC (ATCC, USA) was cultured in RenaEpi Growth Medium (Cell Applications, San Diego). HEK293AD was maintained in DMEM high glucose (HYCLONE), 10% fetal bovine serum (HYCLONE) and 1% MEM non-essential amino acids (Gibco). Cell lines were passaged using 0.25% Trypsin (Pan-Biotec). All cells were routinely tested to be free from mycoplasma using the MycoAlert^TM^ PLUS Mycoplasma Detection Kit (Lonza Bioscience).

### Human subjects and kidney sections

Human adult kidney cryosections were procured from the National Disease Research Interchange (NDRI). Urine samples were collected from Khoo Teck Puat Hospital. The subjects gave informed consent, and the samples were anonymised. The use of these samples was approved by the NHG DSRB 2013/01068.

### Kidney differentiation

hPSC colonies were handpicked into 10 cm plates pre-coated with 0.1% gelatin and MEF media. Once confluent, the hPSC colonies were dissociated with TrypLE (ThermoFisher, USA) and passed through a 40μm filter to obtain single cells. 200,000 cells were seeded into each well of a 6 well plate containing 2 ml of mTeSR1 per well (Stem Cell Technologies, Canada). The attachment efficiency of the cells was enhanced via the addition of Rho-associated, coiled-coil containing protein kinase (ROCK) inhibitor, Y-27632 (1:1000) (Stem Cell Technologies, Canada). hPSCs were subsequently differentiated into kidney organoids following the 28-day kidney differentiation protocol developed by Morizane and colleagues with minimal modifications using AdvRPMI1640 (Sigma, #12633-012) as the basal media [26].

On Day 0 (start of differentiation), hPSCs were cultured in 2 ml AdvRPMI1640 + 8 μM CHIR99021 + 10 μM Noggin (Stem Cell Technologies, Canada). The same culture media was refreshed 2 days after the start of differentiation on Day 2. On Day 4, cells were cultured in 3 ml AdvRPMI1640 + 10 ng/ml Activin A (Stem Cell Technologies, Canada). On Day 7, cells were cultured in 2 ml AdvRPMI1640 + 10 ng/ml FGF9 (Stem Cell Technologies, Canada). On Day 9, cells were dissociated into single cells using TrypLE and seeded into round bottom low attachment 96 well plates (Corning, USA) at a seeding density of 100,000 cells per well. Each kidney organoid was cultured in 200 μl AdvRPMI1640 + 3 μM CHIR99021 + 10 ng/ml FGF9 (Stem Cell Technologies, Canda). On Day 11, cells were cultured in 200 μl AdvRPMI1640 + 10 ng/ml FGF9. Thereafter, the kidney organoids were cultured in 200 μl of basal AdvRPMI1640 media, with a fresh change of media every other day till Day 28.

Cells or organoids at the five time points: Days 0, 9, 14, 21 and 28 were harvested in triplicates for analyses. At time points Days 0 and 9, cells from 1 well of a 6-well plate were harvested as technical replicates. At time points Days 14, 21 and 28, each replicate consisted of minimally eight organoids.

### RT-qPCR analysis

RNA was harvested using the Nucleospin® RNA kit (Macherey-Nagel). Reverse transcription was performed using the High-Capacity cDNA Reverse Transcription Kit (ThermoFisher Scientific). Quantitative polymerase chain reaction (qPCR) was performed using iTaq™ Universal SYBR® Green Supermix (BIO-RAD) with primers listed in Table S3.

### Chromatin Immunoprecipitation (ChIP)

ChIP was performed following our published protocol [27]. Approximately 48–60 kidney organoids were dissociated into smaller cell clumps via incubation with TrypLE (Invitrogen, USA) for 3 min after washing twice with 1xPBS. Thereafter, the cells were cross-linked with 3.3 mg/ml of dimethyl 3,3'-dithiobispropionimidate (DTBP, SIGMA) and 1 mg/ml of 3,3'-dithiodipropionic acid (DSP, SIGMA) for 15 min at room temperature before another round of cross-linking with 1% formaldehyde for 15 min at room temperature. 0.125 M glycine was added to the cells to quench the crosslinking reaction. Ice-cold cell lysis buffer comprising 10 mM Tris-Hcl pH 8, 10 mM NaCl and 0.2% NP-40 was added to lyse the cells. Nuclear lysis was then performed using the nuclear lysis buffer comprising of 50 mM Tris-HCl pH 8, 10 mM EDTA and 1% SDS. Protease inhibitors: 0.1 mM leupeptinhemisulfate, 1 µM pepstatin and 1 mM phenylmethylsulfonyl fluoride were added to the respective lysis buffers fresh prior to lysis. The nuclear lysates were then diluted in IP dilution buffer comprising 20 mM Tris-HCl pH 8, 2 mM EDTA, 150 mM NaCl, 0.01% SDS and 1% Triton X-100. Next, sonication was performed on the Misonix Q500 sonicator (QSonica) for 12 cycles using the following settings: 30 s on/45 s off, 30% power. The sonicated lysate was then pre-cleared using 10 µg of rabbit IgG and protein A/G agarose beads for 3 h at 4 °C before immunoprecipitation. After pre-clearing, 10% (by volume) of the lysate were collected as input for downstream analysis. The remaining lysates were split equally into two portions and incubated with either 10 µg HNF1A antibody (ab96777) or 10 µg rabbit IgG (SantaCruz) overnight at 4 °C. Following, the samples were washed twice with IP wash buffer and Tris-EDTA buffer before the immunoprecipitated genomic DNA (gDNA) was eluted. Elution of gDNA was achieved via incubation with the IP elution buffer comprising of 100 mM NaHCO_3_, 1% SDS, 100 mM DTT. After elution, the samples were treated with RNaseA + 5 M NaCl and Proteinase K to initiate reverse crosslinking and to obtain the immunoprecipitated gDNA. Lastly, HNF1A-bound gDNA was extracted using the phenol/chloroform method.

### ChIP-Seq library preparation and analysis

Library preparation was done using NEB# E7645 NEBNext Ultra II DNA Library Prep Kit for Illumina®. NEXTSEQ High Output was performed using the Illumina NEXTSEQ 500 Sequencers with the Illumina® Reagent v2 (75 cycle kit) Kit. Sequencing adapters were trimmed using BBDuk from the BBMap toolkit (https://sourceforge.net/projects/bbmap). Trimmed reads were aligned to GRCh38 with Bowtie2 and reads non-uniquely mapped were discarded. Filtering of unmapped and duplicate aligned reads were performed using samtools and sambamba. Peaks were called using MACS2 version 2 2.7.1 against input control library with default *q*-value (minimum FDR) cut-off of 0.05. Motif enrichment analysis was performed using the HOMER suite of programs [28]. For the generation of average plot and heatmap, the deepTools package [29] was used to calculate scores based on the read density values of mapped reads. Data reported in this paper were deposited into Gene Expression Omnibus (GEO) accessible via accession number GSE196273.

### Profiling expression pattern of genes in kidney organoids

To characterize the expression pattern of genes, we leveraged on a published scRNA-Seq dataset performed on D26 kidney organoids generated using Morizane’s protocol (GEO: GSM3320185).

Using the expression dot plots at Kidney Integrative Transcriptomics (http://humphreyslab.com/SingleCell/displaycharts.php), we manually scrutinized the expression profile of target genes and categorized them based on their expression pattern. In this analysis, a gene is considered predominantly expressed when it fulfils two criteria. Firstly, the expression of the gene must be confined to either the tubular epithelial cells (PT1/PT2/LH), mesenchymal cells (M1/M2/M3) or podocytes (P1/P2/P3). Gene expression in the neuronal (N1/N2/N3) and muscle (MU) clusters were disregarded as these cell types are part of the off-target population. Secondly, the average gene expression as visualized from the dot plot must be greater than or equal to a normalized score of 1 in the cell cluster where it is predominantly expressed. A consolidated summary of the genes that are predominantly expressed at each of the cell clusters is documented in Table S2.

### Transfection of cells

Transfection was performed using Lipofectamine^TM^ 2000 (ThermoFisher, USA) following manufacturer’s protocol. 2 μg of plasmids were used for overexpression experiments. Primers used to generate sh*HNF1A* constructs are listed in Table S3.

### Immunofluorescence staining

Cryo-sectioning of kidney organoids was performed by the Advanced Molecular Pathology Laboratory (AMPL), A*STAR using the tissue-tek OCT compound. The cryo-embedded cells were sectioned and mounted onto glass slides and stored at −80 °C.

Cells were fixed with 4% paraformaldehyde (Wako, Japan), permeabilized with 0.025% Triton X-100 and blocked with PBS containing 10% donkey serum (Merck, USA) for 1 h at 4 °C. After blocking, the cells were co-stained with primary antibodies overnight at 4 °C. Secondary antibodies were added and incubated at 4 °C for 1 h before staining with 4',6-diamidino-2-phenylindole (DAPI) (Sigma-Aldrich, US) (1:5000) for 20 min. Imaging was done using the Olympus FluoView FV1000 confocal microscope or Opera Phenix® Plus High-Content Screening System (Perkin Elmer).

For quantification of fluorescence intensity, whole images corresponding to the appropriate fluorescence channel were subjected to the ‘Measurement’ function under the ‘Analyze’ tab of ImageJ. The raw values obtained were divided by the values for DAPI intensity to obtain normalized readings. These normalized readings were then used to plot the bar graphs and for subsequent statistical testing. For single cell analysis, the Columbus Image Data Storage and Analysis System was adopted to segment cells and quantify fluorescence intensity of SLC51B. At least two representative images were captured for each condition studied in each independent experiment.

### Immunoblotting

Cells were lysed with the Mammalian Protein Extraction Reagent (M-PER, Thermo Scientific) in the presence of protease and phosphatase inhibitors 2/3 (Sigma). Protein was quantified using the BCA assay (Thermo Scientific). Immunoblotting was performed as previously described [30] using antibodies detailed in Table S4.

### Lentiviral-mediated knockdown of *HNF1A*

pLKO.1-sh*HNF1A* plasmids were packaged into lentiviruses by transfecting HEK293FT cells with pLKO.1-sh*HNF1A* and expression plasmids encoding for rev, VSV-g and gag-pol proteins. Lentiviral particles were collected and concentrated via ultracentrifugation at 23,000 rpm for 90 min at 4 °C. Virus titer was quantified using the Lenti-X p24 Rapid Titer Kit (TakaraBio).

For viral transduction, RPTECs were plated at 250,000 or 500,000 cells for the harvesting of RNA or protein respectively. One day after seeding, RPTECs were transduced with pLKO.1-sh*HNF1A* lentivirus together with 10 μg/ml polybrene (Millipore) for 24 h. RNA and protein were harvested 48 h after transduction.

### E1S ELISA assays

250,000 RPTECs were transduced with lentiviruses for 72 h prior to treatment with 400 μg/ml E1S (Sigma, E9145). 16–24 kidney organoids were collected into a 15 ml falcon tube and treated with 400 μg/ml E1S. Treatment was performed in respective culture media at 37 °C. To quantify remaining E1S, culture media were collected at timepoints: 5, 10, 15 and 60 min after treatment. ELISA was performed using the competitive E1S ELISA kit (Sigma, # EIA17E3S). Total protein was harvested using M-PER and quantified via BCA assay. E1S uptake was calculated using the following formula:

$$\frac{{\left( {400\,\upmu {{{\mathrm{g}}}}/{{{\mathrm{mL}}}}-{{{\mathrm{concentration}}}}\;{{{\mathrm{of}}}}\;{{{\mathrm{E}}}}1{{{\mathrm{S}}}}\;{{{\mathrm{left}}}}\;{{{\mathrm{in}}}}\;{{{\mathrm{supernatant}}}}} \right)}}{{{{{\mathrm{Total}}}}\;{{{\mathrm{protein}}}}\left( {\upmu {{{\mathrm{g}}}}/{{{\mathrm{mL}}}}} \right)}}$$

For quantification of E1S in urine samples, E1S levels were normalized to total urinary creatinine content as measured using a creatinine urinary detection kit (Sigma, EIACUN) according to manufacturer’s protocol.

### Statistical analyses

All data were presented as mean ± SEM unless otherwise stated. For statistical testing, unpaired *t*-tests were performed for analyses involving only two independent groups, while one- or two-way ANOVA followed by Tukey’s multiple comparison test were performed for analyses involving three or more groups. F-test was performed for each dataset to test for normality. Each dot in a bar graph is representative of data obtained from one cell line in an independent experiment. For hPSC related experiments, two WT-hPSC lines (H9 and iAgb) were used, and three independent cell lines were derived from each of the two MODY3 patients recruited. Each independent experiment involving MODY3-hPSCs is comprised of eight cell lines: two WT hiPSCs (H9/iAgb), three iP001 hiPSCs and three iP002 hiPSCs. * represents a *p*-value of <0.05, ** a *p*-value of <0.01 and *** a *p*-value of <0.001. All graphs were generated using either the GraphPad Prism 8 software or ggplot2 package on R. No statistical methods were used to pre-determine sample sizes in our in vitro experiments. Based on means and standard deviations from previous studies with MODY3 patients on urinary CC16 excretion^8^, a power analysis (set at 80%) revealed that <6 human subjects reliably distinguish mutant and control populations for >30% change in urinary metabolite secretion (p < 0.05). Hence, we recruited at least six subjects for each study group in this study.

## Results

### HNF1A is majorly expressed in human renal proximal tubule epithelial cells

**Fig. 1:**
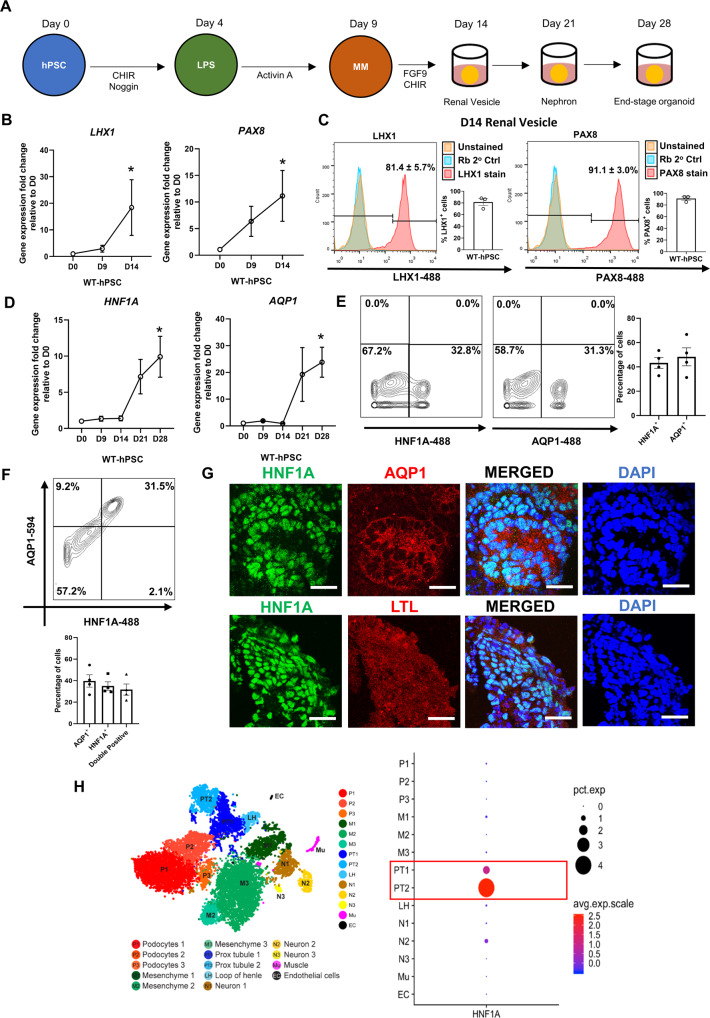
HNF1A is expressed during kidney differentiation and is majorly expressed in the proximal tubules of kidney organoids. **A** Schematic depicting the kidney differentiation protocol adopted to generate end-stage kidney organoids. hPSC: human pluripotent stem cells. LPS: late primitive streak. MM: metanephric mesenchyme. **B** Relative mRNA expression levels of the various renal vesicle markers (*LHX1, PAX8*) at the different time points of kidney differentiation (D0, D9, D14). Error bar represents ± SEM. N = 3 experiments have been performed. *p-value < 0.05. **C** Flow cytometry experiments evaluating the percentage of cells expressing LHX1 or PAX8 at the protein level during D14 of kidney differentiation. Comparison is done relative to an unstained and secondary antibody control. Each dot represents data obtained from one independent cell line during one renal differentiation experiment. **D** Relative mRNA expression level of *HNF1A* and *AQP1* (proximal tubule marker) at the different time points of kidney differentiation (D0, D9, D14, D21, D28). Each dot represents data from one cell line from one independent experiment. Error bar represents ±SEM. *p-value < 0.05. **E** Flow cytometry analysis of HNF1A and AQP1 expression at D28 kidney differentiation. Plot depicts the results obtained from one representative experiment. Bar graph represents the consolidated results across four experiments. Error bar represents ± SEM. **F** Flow cytometry analysis of HNF1A and AQP1 co-expressing cells at D28 of kidney differentiation. Plot depicts the results obtained from one representative experiment. Bar graph represents the consolidated results across four experiments. Error bar represents ±SEM. **G** Immunofluorescence staining of HNF1A, AQP1 and LTL on D28 kidney organoids. *N* = 3 independent experiments have been performed. Scale bar represents 100 µm. **H** Dot plot depicting the gene expression level of *HNF1A* in the various cell clusters found in kidney organoids generated using Morizane protocol. Data is extracted from http://humphreyslab.com/SingleCell/ using publicly available dataset published by Wu et al. [32].

We first examined the expression profile of *HNF1A* during kidney differentiation using an established protocol [31] on two wild-type (WT) hPSC lines (Fig. 1A). Consistent with previous literature [31], qRT-PCR analyses revealed that the transcript expression of renal vesicle markers *LHX1*/*PAX8* increased significantly as differentiation progressed (Fig. 1B), with >80% of the cells being LHX1^+^ or PAX8^+^ by day 14 (Fig. 1C). Thereafter, we evaluated the transcript expression of *HNF1A* and *AQP1*, a proximal tubule marker, during differentiation. Notably, both *HNF1A* and *AQP1* expression increased steadily and peaked by the end of kidney differentiation on D28 (Fig. 1D). Flow cytometry experiments then demonstrated that D28 organoids expressed HNF1A and AQP1 proteins (Fig. 1E), with majority of AQP1^+^ cells co-expressing HNF1A (Fig. 1F). Subsequent immunofluorescence staining on D28 organoid cryosections further confirmed the co-expression of HNF1A and proximal tubule markers AQP1/LTL (Fig. 1G).

To examine the expression pattern of *HNF1A* in human kidney organoids, we leveraged on a publicly-available single-cell RNA-Seq dataset [32]. Single-cell analyses revealed that *HNF1A* is expressed mainly in cells whose transcriptome mimicked that of the proximal tubules (PT1/PT2) (Fig. 1H). Additionally, single-nuclear transcriptomic studies performed on healthy adult human kidney tissue [32, 33] also revealed *HNF1A* to be largely confined to the proximal tubule epithelial cells (Fig. S1A, B). To validate this finding, we performed immunofluorescence staining on the cryosections of three healthy human kidneys and demonstrated that HNF1A is mainly expressed in regions where AQP1 is detected (Fig. S1C); similar to what was observed in our D28 organoids (Fig. 1F, G). Collectively, these results suggest that HNF1A is increasingly expressed during human kidney differentiation, with majority of its expression in the hPSC-derived renal proximal tubule cells and in adult human kidney cells.

### ChIP-Seq and targeted qRT-PCR reveal putative HNF1A target genes in human kidney cells

**Fig. 2:**
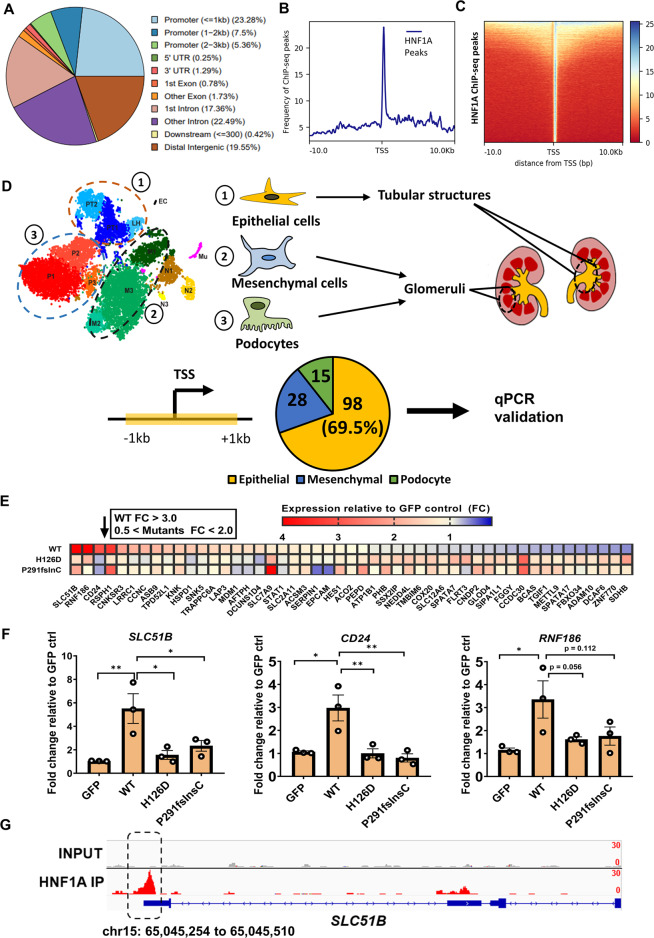
ChIP-Seq and targeted qPCR screen reveals direct gene targets of HNF1A in human kidney cells. **A** Pie chart depicting the enrichment profiles of HNF1A ChIP-Seq performed on D28 kidney organoids derived from WT-hPSCs. **B** Density plot and (**C**) heatmap summarizing the peak count frequency of HNF1A-bound regions that are ±10 kb from the transcriptional start site (TSS). **D** Schematic illustration depicting the strategy adopted to classify and shortlist putative target genes from our ChIP-Seq analysis for the targeted qPCR screen. Only genes whose promoter region (defined as ±1 kb from TSS) were found to be bound by HNF1A were considered. **E** Heatmap depicting the results from our targeted qRT-PCR screen performed on HEK293AD cells overexpressing either WT-HNF1A or the HNF1A mutants. Colors in the heatmap represents the relative expression level in terms of fold-change (calculated by 2^-ΔΔCt^) in the respective conditions as compared to GFP (empty vector transfected) control. **F** qRT-PCR analysis of *SLC51B, CD24* and *RNF186* in HEK293AD cells overexpressing either WT-HNF1A, HNF1A H126D or HNF1A P291fsInsC. Each dot represents data from one independent experiment. Error bar represents ±SEM. *p-value < 0.05, **p-value < 0.01. **G** ChIP-Seq profile of the significantly called peak along the *SLC51B* gene locus. Regions whereby the enrichment is significantly called is denoted in the highlighted box along with the corresponding genomic coordinate labeled.

To investigate the transcriptional targets of HNF1A in the human kidney, we next performed ChIP-Seq on D28 kidney organoids where HNF1A protein could be robustly detected (Fig. 1F, G). ChIP-Seq analysis revealed an enrichment of gene promoters (±3 kb from TSS, comprising 36.14% of all significantly called peaks) that are bound by HNF1A (Fig. 2A). Analysis of the HNF1A binding profile revealed an increased binding frequency at the proximal promoter of genes, as is expected of ChIP-Seq performed on transcription factors (Fig. 2B, C). Altogether, 16,310 HNF1A-bound genomic loci were identified based upon a cut-off FDR score of <0.05 (Table S1).

To ascertain that these enriched peaks are not the result of poor antibody specificity, we validated that the HNF1A antibody (ab96777) used is suitable for ChIP according to guidelines from the ENCODE consortia [34]. To achieve this, we performed immunoblotting and immunofluorescence experiments on HEK293AD cells which has low endogenous HNF1A expression. Minimal non-specific signal was observed in HEK293AD cells transfected with the pCDH-GFP empty vector (Fig. S2A, B). Following the ectopic overexpression (OE) of a FLAG-tagged HNF1A construct, a specific band corresponding to the predicted molecular weight of HNF1A (~67 kDa) could be detected (Fig. S2A). Moreover, a clear immunofluorescence signal that co-localizes with FLAG in the nucleus could be observed (Fig. S2B). Additionally, our laboratory has previously utilized this antibody to perform ChIP-Seq on hPSC-derived islet-like cells and was able to identify the HNF1A binding motif ‘TTAAT’ as the top enriched motif [5]. Similarly, we were able to detect the ‘TTAAT’ motif following motif enrichment analysis on our D28 kidney organoid ChIP-Seq dataset, albeit not the top enriched motif (Fig. S2C). Together, these data confirmed that the HNF1A (ab96777) antibody used is suitable for HNF1A ChIP experiments.

Next, we focused on the 3797 genomic loci that were mapped to within ±1 kb of promoter-TSS of 2800 unique genes as putative HNF1A targets (Table S1). Given that HNF1A expression is confined mainly to the proximal tubule-like cells (Fig. 1H), we reasoned that its direct transcriptional targets are likely to adopt a similar expression pattern. Hence, we evaluated the expression pattern of the 2800 putative targets using the aforementioned scRNA-Seq dataset to arrive at a list of candidate genes. Clustering of end-stage kidney organoids revealed three main cell clusters of the renal lineage, henceforth referred to as [1] tubular epithelial cells (PT1/PT2/LH), [2] mesenchymal cells (M1/M2/M3) and [3] podocytes (P1/P2/P3) [32] (Fig. 2D). Amongst the 2800 putative targets, 141 candidate genes were found to be expressed predominantly in one of the three renal cell types (Fig. 2D and Table S2). Importantly, 98 out of the 141 (~69.5%) cell type-enriched candidate genes were expressed predominantly in the tubular epithelial cells (Fig. 2D), similar to the expression pattern of *HNF1A*, suggesting that these genes could be direct transcriptional targets of HNF1A in the human kidney.

We next performed a targeted qPCR screen to evaluate the expression levels of these candidate genes following the OE of WT-HNF1A or two HNF1A mutants that cause MODY3–H126D [5] and P291fsInsC [9, 35] in HEK293AD cells (Fig. S3A, B). P291fsInsC is the most common form of HNF1A mutation that has been implicated with MODY3 [35], while H126D has been previously characterized as a loss-of-function HNF1A variant [5]. Consistent with the expected loss-of-function effects of MODY3 variants, we saw that the OE of WT but not the two HNF1A mutants resulted in a significant upregulation of three known target genes of HNF1A, namely: *HNF4A* [36]*, UGT2B4* [5, 13] and *OAT3* [37] (Fig. S3C). These three known target genes share similar expression pattern as *HNF1A* and are predominantly expressed in the renal tubular epithelial cells (Fig. S3D).

Having confidence that this was a feasible approach to detect target genes of HNF1A, we next performed a qRT-PCR screen on 49 of the 98 putative gene targets identified (Fig. 2E). Based upon a fold-change cut-off >3 for WT-HNF1A and 0.5 <FC < 2.0 for HNF1A mutant overexpression, we uncovered three new target genes: *SLC51B*, *CD24* and *RNF186* that are upregulated by WT-HNF1A but not the two MODY3 mutants (Fig. 2F). The expression pattern of these genes is confined mainly to the tubular epithelial cells, similar to that for *HNF1A* (Fig. 1H and S3E). Visualization of the HNF1A ChIP-Seq peaks further confirmed an enrichment of reads mapped to the proximal promoter of these genes (Fig. 2G and S2D). Together, these findings demonstrated *SLC51B*, *CD24* and *RNF186* as novel direct transcriptional targets of HNF1A in human renal proximal tubule cells.

### SLC51B expression is decreased in HNF1A mutant and knocked-down human kidney cells

**Fig. 3:**
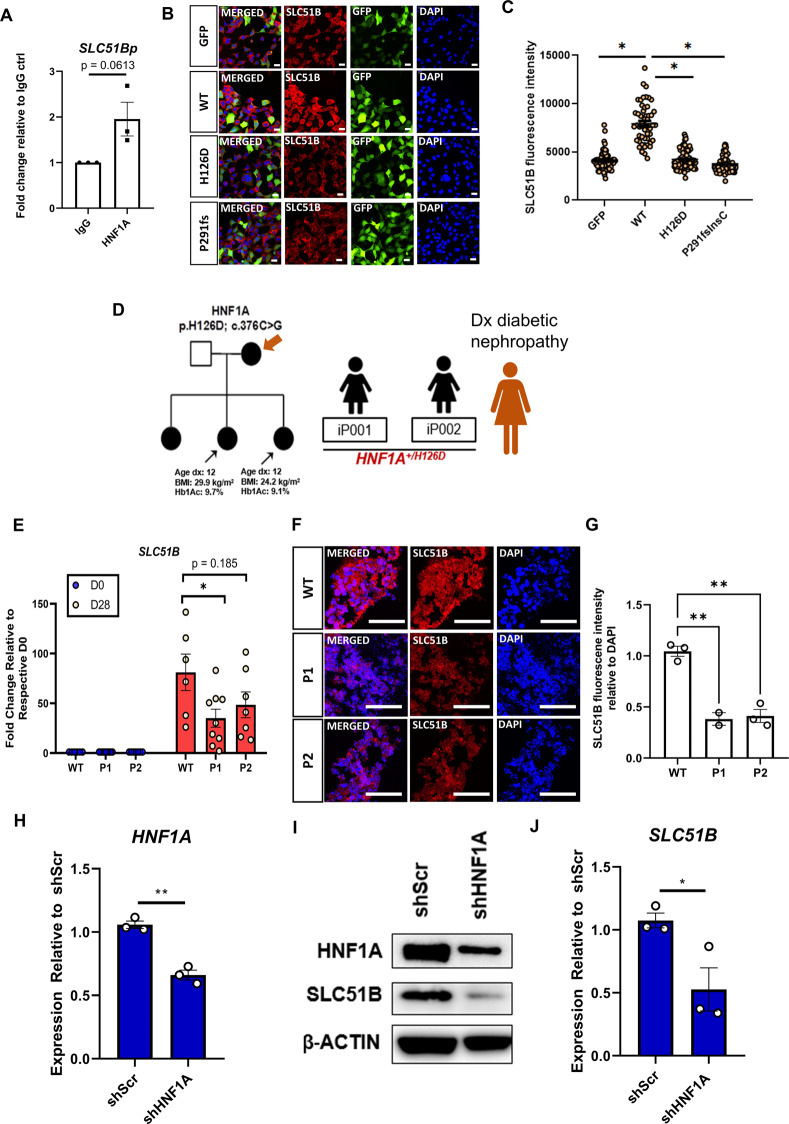
WT-HNF1A regulates the expression level of SLC51B in kidney cells. **A** ChIP-qPCR analysis of HNF1A binding onto the *SLC51B* promoter region in kidney cells. *N* = 3 independent experiments have been performed. **B** Immunofluorescence staining for SLC51B (red), GFP (green) and DAPI (blue) in HEK293AD cells overexpressing either WT-HNF1A or HNF1A mutants. Scale bar represents 20 µm. **C** Quantification of immunofluorescence data from HEK293AD cells overexpressing either WT-HNF1A or HNF1A mutants using Perkin Elmer’s Columbus image analysis system. Each dot represents fluorescence intensity of SLC51B in one GFP^+^ cell. Error bar represents ±SEM. *p-value < 0.05. **D** Family tree of the MODY3 patients recruited in this study. Black arrow indicates the patients recruited in this study. **E** Relative mRNA expression levels of *SLC51B* in MODY3 vs WT kidney organoids. Each dot represents data from one cell line from one independent experiment. Error bar represents ±SEM. *p-value < 0.05. **F** Immunofluorescence staining for SLC51B and DAPI in D28 MODY3 or WT kidney organoids. Scale bar represents 100 µm. **G** Quantification of fluorescent intensity of D28 MODY3 vs WT kidney organoids using ImageJ. Error bar represents ±SEM. *p-value < 0.05. **H** Relative mRNA expression levels of *HNF1A* in RPTEC cells transduced with either pLKO.1-sh*SCR* or pLKO.1-sh*HNF1A* construct. Error bar represents ±SEM. *p-value < 0.05. **I** Western blotting of HNF1A and SLC51B expression levels in sh*SCR* and sh*HNF1A* RPTEC cells. **J** Relative mRNA expression levels of *SLC51B* in RPTEC cells transduced with either pLKO.1-sh*SCR* or pLKO.1-sh*HNF1A* construct. Error bar represents ± SEM. *p-value < 0.05.

Among the three direct transcriptional targets uncovered, *SLC51B* has the strongest link with kidney function, having been implicated with the transportation of conjugated steroids across the basolateral membrane of renal epithelial cells [38]. We first performed ChIP-qPCR to confirm the binding of HNF1A onto the proximal promoter of *SLC51B* (Fig. 3A). Next, we interrogated whether SLC51B protein is differentially regulated by WT-HNF1A and the two MODY3 *HNF1A* mutants. Consistent with qRT-PCR, immunofluorescence staining of HEK293AD cells revealed that HNF1A H126D and P291fsInsC exhibited diminished ability to upregulate SLC51B protein as compared to WT-HNF1A (Fig. 3B, C).

We then leveraged on hiPSCs (iP001 and iP002) derived from two MODY3 female siblings harboring the *HNF1A*^*+/H126D*^ mutation [5] (Fig. 3D). Notably, the mother of these two siblings was diagnosed with diabetic nephropathy [39, 40]. MODY3 hiPSC-derived kidney organoids did not differ morphologically from WT kidney organoids (Fig. S4A). Additionally, the transcript expression of renal genes such as *OSR1* (mesodermal marker), *PAX8* (renal vesicle marker)*, AQP1* (proximal tubule marker) and *HNF1A* were comparable to WT organoids throughout differentiation and at D28 (Fig. S4B), indicating that the *HNF1A*^*+/H126D*^ mutation does not affect the propensity of hPSCs to differentiate into kidney organoids. Importantly, MODY3 hiPSC-derived kidney organoids exhibited diminished ability to upregulate *SLC51B* gene expression during differentiation (Fig. 3E), resulting in lowered SLC51B protein expression (Fig. 3F, G). To confirm that *SLC51B* is directly regulated by HNF1A in the renal proximal tubule cells, we knocked down *HNF1A* in the human primary renal proximal tubule epithelial cells (RPTECs) via lentiviral transduction (Fig. 3H, I). Knockdown of HNF1A resulted in a significant decrease in the transcript and protein expression of *SLC51B* (Fig. 3I, J). Together, these findings established *SLC51B* as a direct transcriptional target of HNF1A in the human proximal tubule cells.

### HNF1A depletion in proximal tubule cells results in defective reabsorption of estrone sulfate (E1S)

**Fig. 4:**
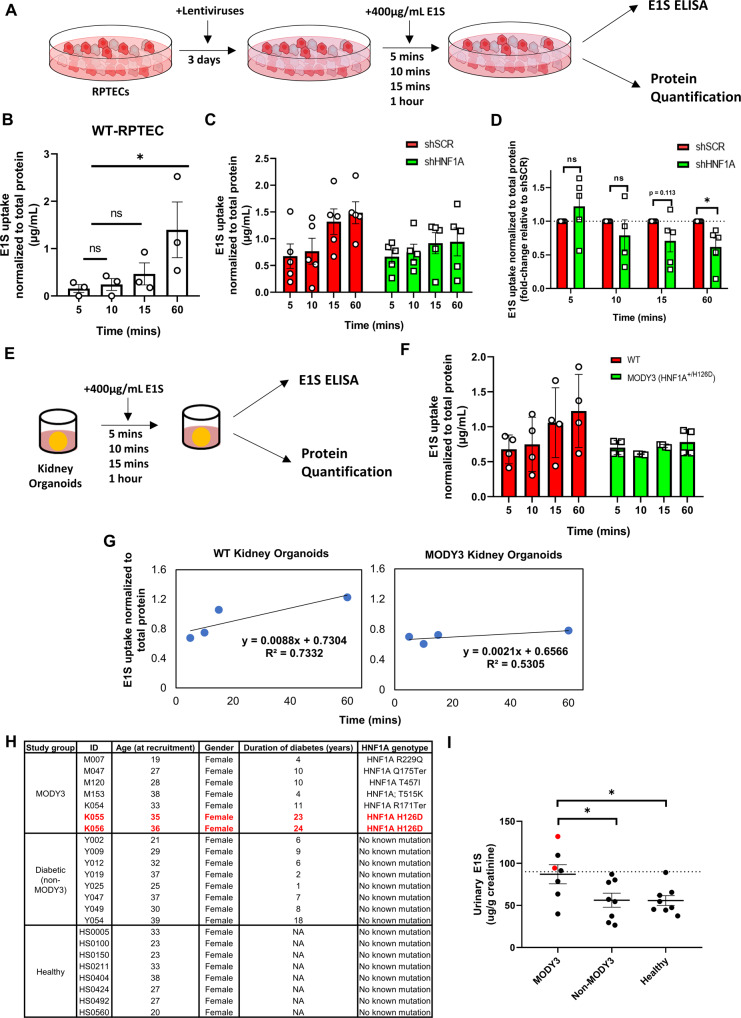
Depletion of HNF1A impedes the uptake of E1S in renal proximal tubule cells. **A** Schematic depicting the strategy adopted for E1S ELISA assay to evaluate estrone sulfate uptake in vitro for RPTEC cells. **B** E1S uptake in wild-type RPTECs over a time course of 60 min. Error bar represents ± SEM. *p-value < 0.05. **C** E1S uptake in sh*SCR* and sh*HNF1A* RPTECs. **D** E1S uptake by fold-change (normalized to sh*SCR* control at respective timepoints) in sh*SCR* and sh*HNF1A* RPTECs. Independent t-tests were performed to compute for *p*-value within each timepoint tested. *p-value < 0.05. **E** Schematic depicting the strategy adopted for E1S ELISA assay to evaluate estrone sulfate uptake in vitro for kidney organoids. A minimum of 16 kidney organoids were collected for treatment in each condition tested. **F** E1S uptake in MODY3 and WT kidney organoids. Error bar represents ±SEM. **G** Linear regression test performed on E1S uptake in WT (left) and MODY3 (right) kidney organoids using the trendline function in Microsoft Excel. **H** Table summarizing the demographics of the seven MODY3, eight non-MODY3 diabetes and eight non-diabetes patients recruited for the study. Details highlighted in red denote the two MODY3 patients used in our kidney organoid studies. **I** Urinary E1S concentration of the patients recruited for this study. Concentration is presented as E1S/creatinine levels (μg/g). Red dots indicate the urinary E1S concentration of the two MODY3 patient utilized in our kidney organoid studies. Statistical analyses were performed using one-way ANOVA followed by Dunnett’s multiple comparisons test.

SLC51B acts in concert with SLC51A to form the OSTα–OSTβ heterodimer transporter complex [38]. Importantly, *Slc51a*^*-/-*^ mice display altered disposition of estrone sulfate (E1S) in the kidneys [41]. To evaluate whether the depletion of HNF1A in RPTECs is sufficient to impede the reabsorption of E1S into the proximal tubules, we treated sh*HNF1A* RPTECs with 400 µg/ml of E1S over a time course up to 60 min (Fig. 4A). ELISA assays showed that E1S is increasingly depleted in the culture media of WT-RPTECs overtime, indicating that the WT-RPTECs were able to uptake E1S (Fig. 4B). Vitally, the depletion of *HNF1A* resulted in a significant reduction in E1S that can be reabsorbed into the RPTECs at 60 min (Fig. 4C, D). To then elucidate whether MODY3 patients with *HNF1A* mutation also present defects in the uptake of E1S at the proximal tubules, we performed the same assay on D28 kidney organoids derived from either WT or MODY3 *HNF1A*^*+/H126D*^ hPSCs (Fig. 4E). Our results confirmed that MODY3 kidney organoids have diminished ability to uptake E1S (Fig. 4F, G). We next evaluated whether the loss of *SLC51B* in MODY3 patients results in an increase in urinary E1S excreted. To do so, we collected urine samples from eight Singaporean non-diabetes (healthy), eight diabetes (non-MODY3) and seven MODY3 patients (Fig. 4H and Table S5). Recruitment was restricted to female participants who were not receiving hormonal therapy and were below the age of 40 as E1S levels were previously reported to vary under such conditions [42]. Consistent with our hypothesis, urinary E1S was significantly higher in the MODY3 group as compared to both non-diabetes and non-MODY3 diabetes patients (Fig. 4I), confirming that more E1S is excreted from the kidneys of MODY3 patients harboring HNF1A loss-of-function mutations.

## Discussion

MODY3 patients with heterozygous *HNF1A* gene mutations tend to manifest renal complications such as diabetic nephropathy, and a reduced renal threshold for glucose, amino acids and proteins [8, 19, 43]. However, besides these clinical reports, little is known about the role and transcriptional targets of HNF1A in the human kidney. In this study, we leveraged upon several human kidney cell models to reveal the biological role and transcriptional targets of HNF1A in human kidney cells.

*HNF1A* expression was found to be upregulated during human kidney differentiation and also abundantly expressed in adult human kidney cells. In particular, a combination of scRNA-Seq, flow cytometry and immunostaining analyses confirmed that HNF1A is predominantly expressed in human renal epithelial cells that co-express the proximal tubule markers AQP1/LTL, consistent with the expression pattern of *Hnf1a* in mouse kidney [44]. Additionally, our ChIP-Seq analyses revealed that a majority of HNF1A-bound promoters (~69.5%) belonged to genes which were predominantly expressed in proximal tubule epithelial cells. This supports the role of HNF1A in contributing to a decline in renal proximal tubule function [8, 15, 19] when its expression is perturbed or lost.

Based on our targeted qRT-PCR screen involving WT and two MODY3 HNF1A mutants, we identified three credible HNF1A transcriptional targets predominantly expressed in renal tubule epithelial cells: *SLC51B*, *CD24* and *RNF186*. To the best of our knowledge, these three target genes have not been previously identified as bound targets of HNF1A. CD24 was previously reported to be upregulated in proximal tubule epithelia upon injury [45]. It was proposed that the induction of CD24, along with vimentin/CD133/kidney-injury-molecule-1, is a key characteristic of dedifferentiated epithelial cells that directly participates in proximal tubule repair [46]. Whether the elevation of CD24 expression is essential for renal tubule repair remains unknown [47]. As HNF1A mutant renal proximal tubule cells are likely to have decreased *CD24* expression, it may be interesting to investigate whether MODY3 patients retain the ability to undergo tubular repair upon renal injury. Separately, RNF186 is a E3 ring-finger ligase that regulates ER stress-regulated apoptosis [48]. Single nucleotide polymorphisms (SNPs) at the *RNF186* locus (p.R179X) have been directly linked to elevated serum creatinine levels and chronic kidney disease in a genome-wide association study conducted on Icelandic individuals [49], and *RNF186* R179X has been characterized as a loss-of-function mutation [50]. These studies imply that the loss of functional RNF186 at the systemic level may predispose individuals to chronic kidney diseases, although it is unknown whether the loss of RNF186 in the proximal tubules can abrogate kidney function.

SLC51B acts in concert with SLC51A to form a heterodimer transporter complex [38]. Similar to HNF1A, both SLC51A and SLC51B were found to be expressed in the liver, intestines, and kidneys [51]. Whether HNF1A mediates the expression of SLC51B in the liver and intestines remains to be validated. *Slc51a*^*-/-*^ mice display increased urinary excretion of conjugated steroids such as E1S, indicating that the OSTα–OSTβ complex plays an essential role in transporting conjugated steroids across the basolateral membrane of renal epithelial cells [41]. Moreover, ectopic overexpression of *Slc51a* and *Slc51b* is sufficient to induce E1S uptake in xenopus oocytes [52]. In our study, we established that HNF1A directly binds to the proximal promoter of *SLC51B* and activates its gene expression. The loss of HNF1A in RPTECs and MODY3 hiPSC-derived kidney organoids resulted in decreased SLC51B expression that accounted for reduced ability to uptake E1S, thus leading to increased urinary E1S excretion from the kidneys.

Physiologically, E1S is formed from the conjugation of estrogen with sulfuric acid and acts as the main source of estrogen storage in the human body [53]. Hence, an impeded ability to reabsorb E1S, leading to increased urinary E1S excretion, may account for a dwindling pool of estrogen available. In this regard, estrogen is known to play nephroprotective roles in the kidney and the administration of estradiol-17β is able to reverse renal injury in various rodent models, including in db/db mice [25], Alb/TGF-beta1 transgenic mice [23], ICER-transgenic male mice [54], and in STZ-induced rats [24]. Thus, the reduced ability to reabsorb E1S may possibly diminish nephroprotection by estrogen and estradiol, in patients with *HNF1A* gene mutations.

MODY3 patients have a higher tendency to develop diabetic nephropathy [17, 55]. However, it remains poorly understood whether renal dysfunction in MODY3 patients occurs primarily due to prolonged exposure to hyperglycemia or the direct effects of *HNF1A* mutation contributing to renal dysfunction. A recent study has reported that MODY3 patients exhibit reduced ability to drive endocytosis at the proximal tubules, owning to a decreased expression of megalin and cubulin at the epithelial cells, leading to proteinuria [8]. Here, we demonstrate that the loss and/or mutation in *HNF1A* reduced the ability of proximal tubule cells to reabsorb E1S due to a decreased expression of SLC51B protein.

Overall, our study provided new insights into the role and transcriptional targets of HNF1A in various human kidney cell models. We demonstrate that the loss of HNF1A expression can have a direct impact on human renal proximal tubule function, leading to an increased urinary excretion of nephroprotective E1S. Moving forward, it would be of interest to investigate whether modulation of E1S uptake and/or administration of estradiol may be utilized to mitigate renal deficiencies that arise as a result of *HNF1A* loss-of-function effects in MODY3 patients.

### Significance statement

Heterozygous mutation(s) in *HNF1A* gene results in maturity onset diabetes of the young 3 (MODY3). These patients have a higher incidence of renal complications. However, the role of *HNF1A* in human kidney cells remain unclear. Here, we demonstrate that the loss and/or mutation of *HNF1A* in human renal proximal tubule epithelial cells resulted in decreased SLC51B-mediated estrone sulfate (E1S) uptake. As E1S is the main source of nephroprotective estradiol in humans, decreased E1S uptake may partly account for renal deficiencies in patients with *HNF1A* gene mutations. Hence, modulation of E1S uptake may be explored to mitigate *HNF1A* loss-of-function effects that can contribute to renal deficiencies.

## Supplementary information

Supplemental Material

Figure S1

Figure S2

Figure S3

Figure S4

Table S1

Table S2

Table S3

Table S4

Table S5

Reproducibility Checklist

Raw WB Images

Ethics Approval Letter

Informed Consent Form

## Data Availability

The data supporting the findings of this study are openly available in GEO repository under ID: GSE196273.

## References

[1] Baumhueter S, Mendel DB, Conley PB, Kuo CJ, Turk C, Graves MK (1990). HNF-1 shares three sequence motifs with the POU domain proteins and is identical to LF-B1 and APF. Genes Dev.

[2] Lau HH, Ng NHJ, Loo LSW, Jasmen JB, Teo AKK (2018). The molecular functions of hepatocyte nuclear factors—in and beyond the liver. J Hepatol.

[3] Byrne MM, Sturis J, Menzel S, Yamagata K, Fajans SS, Dronsfield MJ (1996). Altered insulin secretory responses to glucose in diabetic and nondiabetic subjects with mutations in the diabetes susceptibility gene MODY3 on chromosome 12. Diabetes..

[4] Yamagata K, Oda N, Kaisaki PJ, Menzel S, Furuta H, Vaxillaire M (1996). Mutations in the hepatocyte nuclear factor-1α gene in maturity-onset diabetes of the young (MODY3). Nature..

[5] Low BSJ, Lim CS, Ding SSL, Tan YS, Ng NHJ, Krishnan VG (2021). Decreased GLUT2 and glucose uptake contribute to insulin secretion defects in MODY3/HNF1A hiPSC-derived mutant β cells. Nat Commun.

[6] Shih DQ, Bussen M, Sehayek E, Ananthanarayanan M, Shneider BL, Suchy FJ (2001). Hepatocyte nuclear factor-1α is an essential regulator of bile acid and plasma cholesterol metabolism. Nat Genet.

[7] Pontoglio M, Barra J, Hadchouel M, Doyen A, Kress C, Bach JP (1996). Hepatocyte nuclear factor 1 inactivation results in hepatic dysfunction, phenylketonuria, and renal Fanconi syndrome. Cell..

[8] Terryn S, Tanaka K, Lengelé JP, Olinger E, Dubois-Laforgue D, Garbay S (2016). Tubular proteinuria in patients with HNF1α mutations: HNF1α drives endocytosis in the proximal tubule. Kidney Int.

[9] Wang H (2000). Molecular targets of a human HNF1alpha mutation responsible for pancreatic beta-cell dysfunction. EMBO J.

[10] Okita K, Yang Q, Yamagata K, Hangenfeldt KA, Miyagawa J, Kajimoto Y (1999). Human insulin gene is a target gene of hepatocyte nuclear factor-1alpha (HNF-1alpha) and HNF-1beta 87. Biochem Biophys Res Commun.

[11] Gautier-Stein A, Zitoun C, Lalli E, Mithieux G, Rajas F (2006). Transcriptional regulation of the glucose-6-phosphatase gene by cAMP/vasoactive intestinal peptide in the intestine: Role of HNF4α, CREM, HNF1α, and C/EBPα. J Biol Chem.

[12] Odom DT, Zizlsperger H, Gordon DB, Bell GW, Rinaldi NJ, Murray HL (2004). Control of pancreas and liver gene expression by HNF transcription factors. Science.

[13] Servitja J-M, Pignatelli M, Maestro MA, Cardalda C, Boj SF, Lozano J (2009). Hnf1α (MODY3) controls tissue-specific transcriptional programs and exerts opposed effects on cell growth in pancreatic islets and the liver. Mol Cell Biol.

[14] Cha J-Y, Kim H, Kim K-S, Hur M-W, Ahn Y (2000). Identification of transacting factors responsible for the tissue-specific expression of human glucose transporter type 2 isoform Gene. J Biol Chem.

[15] Pontoglio M, Prié D, Cheret C, Doyen A, Leroy C, Froguel P (2000). HNF1α controls renal glucose reabsorption in mouse and man. EMBO Rep.

[16] Maher JM, Slitt AL, Callaghan TN, Cheng X, Cheung C, Gonzalez FJ (2006). Alterations in transporter expression in liver, kidney, and duodenum after targeted disruption of the transcription factor HNF1α. Biochem Pharmacol.

[17] Poitou C, Francois H, Bellanne-Chantelot C, Noel C, Jacquet A, Clauin S (2012). Maturity onset diabetes of the young: Clinical characteristics and outcome after kidney and pancreas transplantation in MODY3 and RCAD patients: a single center experience. Transpl Int.

[18] Isomaa B, Henricsson M, Lehto M, Forsblom C, Karanko S, Sarelin L (1998). Chronic diabetic complications in patients with MODY3 diabetes. Diabetologia..

[19] Menzel R, Kaisaki PJ, Rjasanowski I, Heinke P, Kerner W, Menzel S (1998). A low renal threshold for glucose in diabetic patients with a mutation in the hepatocyte nuclear factor-1α (HNF-1α) gene. Diabet Med.

[20] Pontoglio M, Sreenan S, Roe M, Pugh W, Ostrega D, Doyen A (1998). Defective insulin secretion in hepatocyte nuclear factor 1α-deficient mice. J Clin Invest.

[21] De Caestecker M, Humphreys BD, Liu KD, Fissell WH, Cerda J, Nolin TD (2015). Bridging translation by improving preclinical study design in AKI. J Am Soc Nephrol.

[22] Christy NP, Shaver JC (1974). Estrogens and the kidney. Kidney Int.

[23] Blush J, Lei J, Ju W, Silbiger S, Pullman J, Neugarten J (2004). Estradiol reverses renal injury in Alb/TGF-β1 transgenic mice. Kidney Int.

[24] Mankhey RW, Bhatti F, Maric C (2005). 17β-Estradiol replacement improves renal function and pathology associated with diabetic nephropathy. Am J Physiol Renal Physiol.

[25] Catanuto P, Doublier S, Lupia E, Fornoni A, Berho M, Karl M (2009). 17 Β-Estradiol and tamoxifen upregulate estrogen receptor Β expression and control podocyte signaling pathways in a model of type 2 diabetes. Kidney Int [Internet].

[26] Morizane R, Bonventre JV (2017). Generation of nephron progenitor cells and kidney organoids from human pluripotent stem cells. Nat Protoc.

[27] Tan WX, Bok CM, Ng NHJTA (2022). Chromatin immunoprecipitation in human pluripotent stem cell-derived 3D organoids to analyze DNA-proteininteractions. Methods Mol Biol.

[28] Heinz S, Benner C, Spann N, Bertolino E, Lin YC, Laslo P (2010). Simple combinations of lineage-determining transcription factors prime cis-regulatory elements required for macrophage and B cell identities. Mol Cell.

[29] Ramirez F, Ryan DP, Gruning B, Bhardwaj V, Kilpert F, Richter AS (2016). deepTools2: a next generation web server for deep-sequencing data analysis | Nucleic Acids Research | Oxford Academic. Nucleic Acids Res.

[30] Loo LSW, Soetedjo AAP, Lau HH, Ng NHJ, Ghosh S, Nguyen L (2020). BCL-xL/BCL2L1 is a critical anti-apoptotic protein that promotes the survival of differentiating pancreatic cells from human pluripotent stem cells. Cell Death Dis.

[31] Morizane R, Lam AQ, Freedman BS, Kishi S, Valerius MT, Bonventre JV (2015). Nephron organoids derived from human pluripotent stem cells model kidney development and injury. Nat Biotechnol.

[32] Wu H, Uchimura K, Donnelly EL, Kirita Y, Morris SA, Humphreys BD (2018). Comparative analysis and refinement of human PSC-derived kidney organoid differentiation with single-cell transcriptomics. Cell Stem Cell.

[33] Muto Y, Wilson PC, Ledru N, Wu H, Dimke H, Waikar SS (2021). Single cell transcriptional and chromatin accessibility profiling redefine cellular heterogeneity in the adult human kidney. Nat Commun.

[34] Landt SG, Marinov GK, Kundaje A, Kheradpour P, Pauli F, Batzoglou S (2012). ChIP-seq guidelines and practices of the ENCODE and modENCODE consortia. Genome Res.

[35] Yamagata K, Yang Q, Yamamoto K, Iwahashi H, Miyagawa J, Okita K (1998). Mutation P291fsinsC in the transcription factor hepatocyte nuclear factor-1α is dominant negative. Diabetes..

[36] Haliyur R, Tong X, Sanyoura M, Shrestha S, Lindner J, Saunders DC (2019). Human islets expressing HNF1A variant have defective β cell transcriptional regulatory networks. J Clin Invest.

[37] Martovetsky G, Tee JB, Nigam SK (2013). Hepatocyte nuclear factors 4α and 1α regulate kidney developmental expression of drug-metabolizing enzymes and drug transporters. Mol Pharmacol.

[38] Ballatori N, Christian WV, Wheeler SG, Hammond CL (2013). The heteromeric organic solute transporter, OSTα-OSTβ/SLC51: a transporter for steroid-derived molecules. Mol Aspects of Med.

[39] Ang SF, Lim SC, Tan C, Fong JC, Kon WY, Lian JX (2016). A preliminary study to evaluate the strategy of combining clinical criteria and next generation sequencing (NGS) for the identification of monogenic diabetes among multi-ethnic Asians. Diabetes Res Clin Pract.

[40] Tan CSH, Ang SF, Lim SC. Response to multiple glucose-lowering agents in a sib-pair with a novel HNF1α (MODY3) variant. Eur J Hum Genet. 2019. 10.1038/s41431-019-0561-8. 10.1038/s41431-019-0561-8 PMC7080716 31844173

[41] Ballatori N, Fang F, Christian WV, Li N, Hammond CL (2008). Ostα-Ostβ is required for bile acid and conjugated steroid disposition in the intestine, kidney, and liver. Am J Physiol Gastrointest Liver Physiol.

[42] Ruder HJ, Loriaux L, Lipsett MB (1972). Estrone sulfate: production rate and metabolism in man. J Clin Invest.

[43] Bingham C, Ellard S, Nicholls AJ, Pennock CA, Allen J, James AJ (2001). The generalized aminoaciduria seen in patients with hepatocyte nuclear factor-1 alpha mutations is a feature of all patients with diabetes and is associated with glucosuria. Diabetes..

[44] Lazzarro D, De Simone V, De Magistris L, Lehtonen E, Cortese R (1992). LFB1 and LFB3 homeoproteins are sequentially expressed during kidney development. Development..

[45] Kusaba T, Lalli M, Kramann R, Kobayashi A, Humphreys BD (2014). Differentiated kidney epithelial cells repair injured proximal tubule. Proc Natl Acad Sci USA.

[46] Kramann R, Kusaba T, Humphreys BD (2015). Who regenerates the kidney tubule?. Nephrol Dial Transplant.

[47] Kusaba T, Humphreys BD (2014). Controversies on the origin of proliferating epithelial cells after kidney injury. Pediatr Nephrol.

[48] Wang P, Wu Y, Li Y, Zheng J, Tang J (2013). A novel RING fi nger E3 ligase RNF186 regulate ER stress-mediated apoptosis through interaction with BNip1. Cell Signal.

[49] Sveinbjornsson G, Mikaelsdottir E, Palsson R, Indridason OS, Holm H, Jonasdottir A (2014). Rare mutations associating with serum creatinine and chronic kidney disease. Hum Mol Genet.

[50] Rivas MA, Graham D, Sulem P, Stevens C, Desch AN, Goyette P (2016). A protein-truncating R179X variant in RNF186 confers protection against ulcerative colitis. Nat Commun.

[51] Ballatori N, Christian WV, Lee JY, Dawson PA, Soroka CJ, Boyer JL (2005). Liver: biology and pathobiology. Hepatology..

[52] Wang W, Seward DJ, Li L, Boyer JL, Ballatori N (2001). Expression cloning of two genes that together mediate organic solute and steroid transport in the liver of a marine vertebrate. Proc Natl Acad Sci USA.

[53] Kuhl H (2005). Pharmacology of estrogens and progestogens: influence of different routes of administration. Climacteric..

[54] Inada A, Inada O, Fujii NL, Nagafuchi S, Katsuta H. Adjusting the 17 b–Estradiol-to-Androgen Ratio Ameliorates Diabetic Nephropathy. J Am Soc Nephrol. 2016. 10.1681/ASN.2015070741. 10.1681/ASN.2015070741 PMC5042662 26940099

[55] Timsit J, Bellanné-Chantelot C, Dubois-Laforgue D, Velho G (2005). Diagnosis and management of maturity-onset diabetes of the young. Treat Endocrinol.

