# Supplementary material for: HNF1A binds and regulates the expression of SLC51B to facilitate the uptake of estrone sulfate in human renal proximal tubule epithelial cells
Source: Cell Death Dis. 2023 May 3;14(5):302. doi: 10.1038/s41419-023-05827-8 (PMC10156747; doi:10.1038/s41419-023-05827-8)
Supplement: Supplementary file 1 — Supplemental Material [file 41419_2023_5827_MOESM1_ESM.docx]

**Supplementary Material**

**Supplementary Figure Legends**

**Figure S1.** **HNF1A is expressed in the human kidney, with major expression in proximal tubule cells.** (A) UMAP plot depicting the expression profile of *HNF1A* in the adult kidney. snRNA-seq was performed on five healthy adult kidney samples. Dataset is available from (33). PT, proximal tubule; PT_VCAM1,
subpopulation of proximal tubule with VCAM1 expression; PEC, parietal epithelial cells; TAL, thick ascending limb; DCT, distal convoluted tubule; CNT,
connecting tubule; PC, principle cells, ICA, Type A intercalated cells; ICB, Type B intercalated cells; PODO, podocyte; ENDO, endothelial cells; MES,
mesangial cells, FIB, fibroblasts; LEUK, leukocytes. (B) tSNE plot depicting the expression profile of *HNF1A* in the adult human kidney of a 62-year-old white male. Dataset is available from (32). (C) Immunohistochemistry staining of HNF1A (red) and AQP1 (green) in three healthy adult kidney cryosections. Scale bar represents 70µm. All single nuclear data was extracted from: <http://humphreyslab.com/SingleCell/>.

**Figure S2. Validation of HNF1A antibody for ChIP experiments.** (A) Immunoblotting of HNF1A in HEK293AD cells transfected with pCDH-GFP-empty-vector or pCDH-1xFLAG-WT-HNF1A. (B) Immunofluorescence staining for HNF1A (red), FLAG/GFP (green) and DAPI (blue) in the various HEK293AD cells. White arrows denote exogenous HNF1A that is found to co-localize with FLAG in the nucleus. (C) Consensus motif recognized in ChIP-Seq data set via HOMER analysis. (D) ChIP-Seq profile of the significantly called peak along the *CD24* and *RNF186* gene loci. Regions whereby the enrichment is significantly called is denoted in the highlighted box along with the corresponding genomic coordinate labeled.

**Figure S3. ChIP experiments reveal transcriptional targets of HNF1A in proximal tubule cells of kidney organoids.** (A) Relative mRNA expression levels of *HNF1A* in HEK293AD cells transfected with either pCDH-WT-HNF1A, pCDH-HNF1A-H126D or pCDH-HNF1A-P291fsInsC for overexpression studies. Each dot represents data from one independent experiment. Error bar represents ± SEM. * indicate p-value <0.05, ** indicate p-value <0.01. (B) Immunoblotting of HNF1A in the HEK293AD cells overexpressing either WT-HNF1A or HNF1A mutants. (C) Relative mRNA expression levels of known HNF1A target genes: *HNF4A*, *UGT2B4* and *OAT3* in the various HEK293AD cells. Each dot represents data from one independent experiment. Error bar represents ± SEM. * indicate p-value <0.05. (D) Dot plot depicting the average expression level and gene expression pattern of *HNF4A*, *UGT2B4* and *OAT3* in end-stage kidney organoids generated from WT-hPSC (H9). (E) Dot plots depicting the average expression level and gene expression pattern of *SLC51B*, *CD24 iso a* and *RNF186* in end-stage kidney generated from Morizane protocol. Data is extracted from <http://humphreyslab.com/SingleCell/>.

**Figure S4. MODY3 and WT-hPSCs exhibit similar propensity to differentiate into kidney organoids.** (A) Bright field microscopy images of WT and MODY3 cells undergoing directed differentiation into kidney organoids at the various timepoints. Scale bar represents 200µm. (B) qRT-PCR analyses depicting the time course expression levels of *OSR1, PAX8, AQP1* and *HNF1A* in both MODY3 and WT cells during kidney directed differentiation. Statistical testing was done comparing the expression levels of the respective genes at D28 in iP001 or iP002 to WT kidney organoids. ns indicate p-value >0.05.

**Supplementary Table Legends**

**Table S1. HNF1A ChIP-Seq on D28 kidney organoids.**

**Table S2. Candidate HNF1A targets expressed in renal cell types.**

**Table S3. List of primers used.**

**Table S4. Antibody resource table.**

**Table S5. Clinical parameters of patients recruited.**
