# Supplementary figures and images for: HNF1A binds and regulates the expression of SLC51B to facilitate the uptake of estrone sulfate in human renal proximal tubule epithelial cells
Source: Cell Death Dis. 2023 May 3;14(5):302. doi: 10.1038/s41419-023-05827-8 (PMC10156747; doi:10.1038/s41419-023-05827-8)

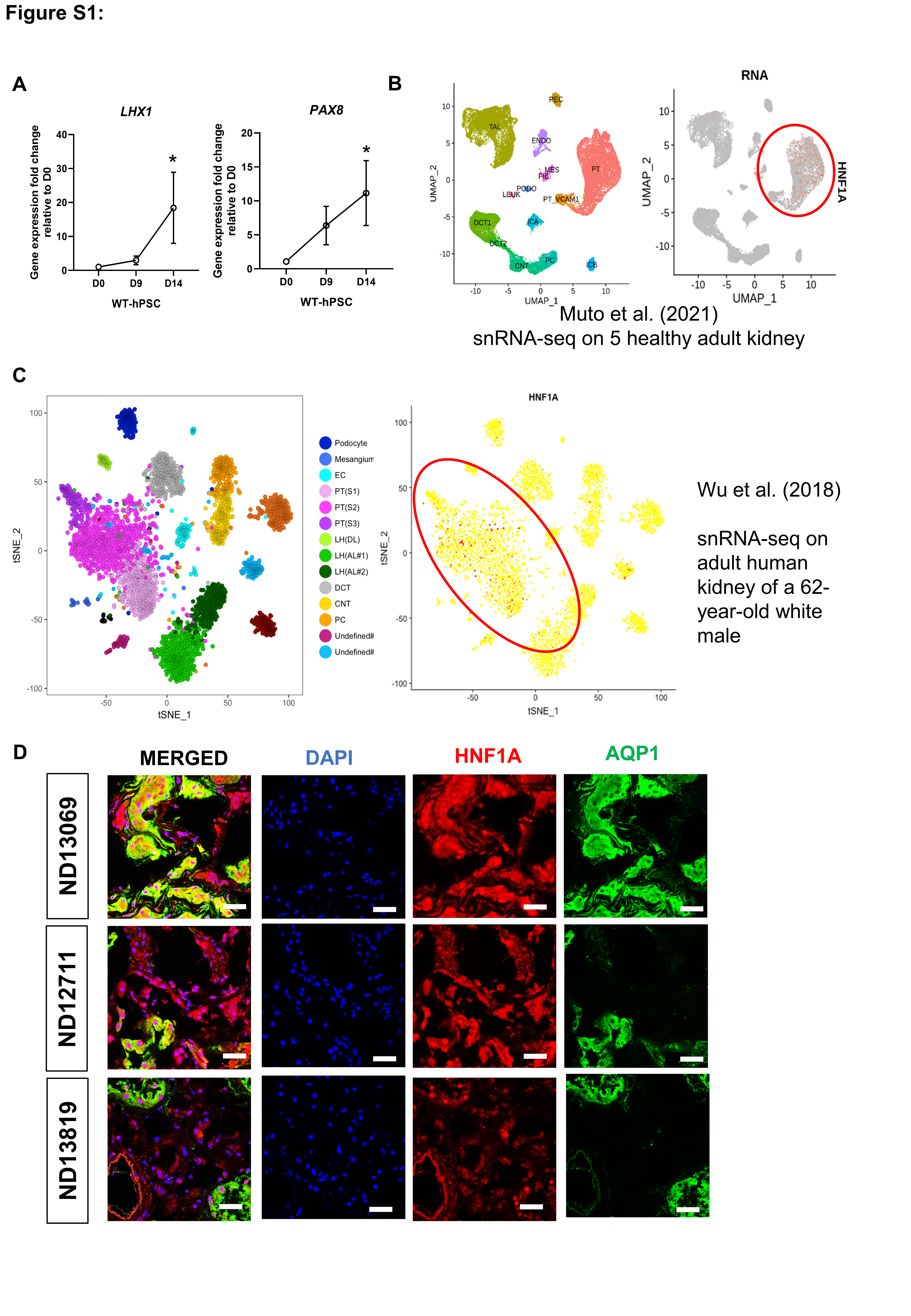

Supplement: Supplementary file 2 — Figure S1 [file 41419_2023_5827_MOESM2_ESM.tif]

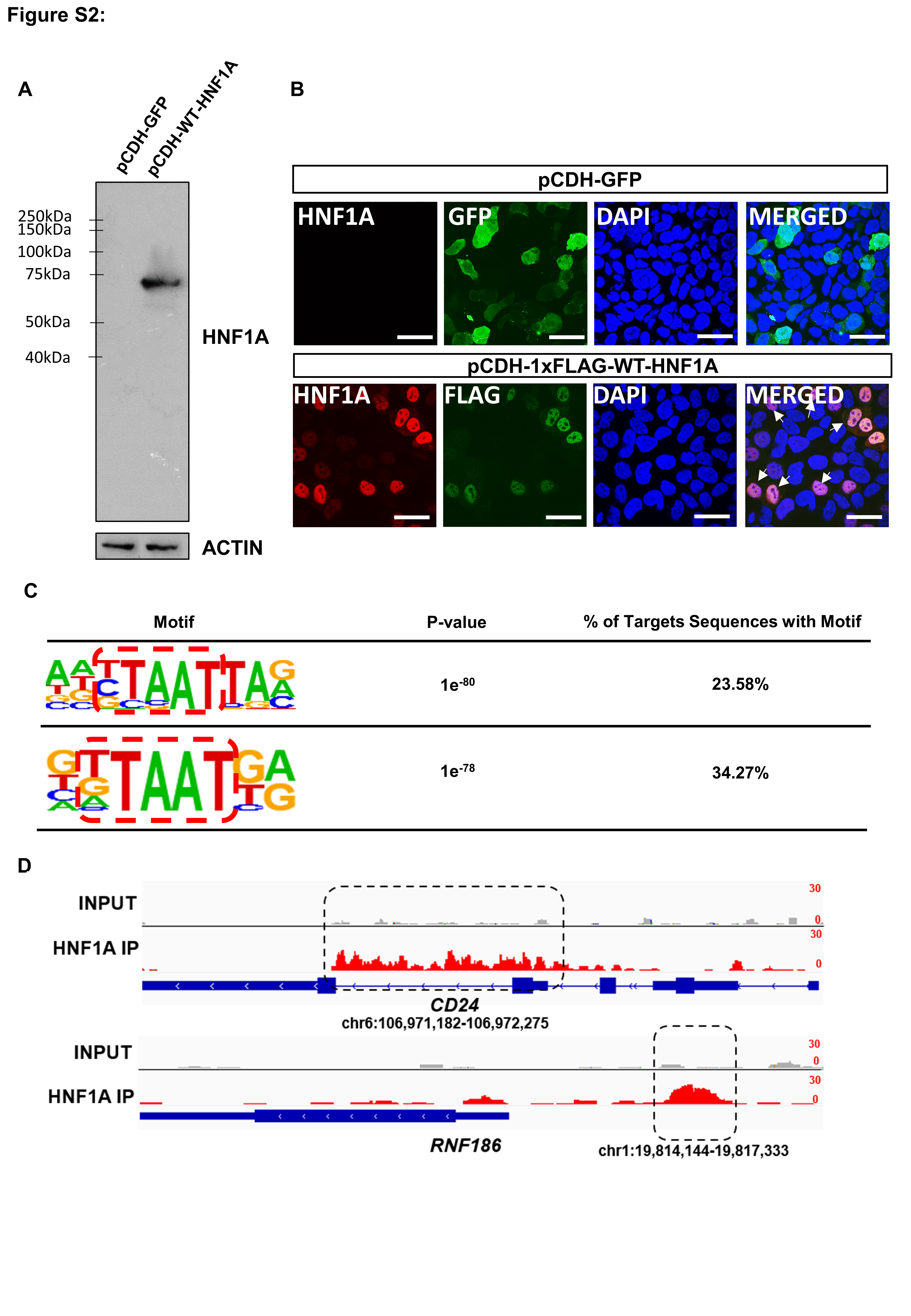

Supplement: Supplementary file 3 — Figure S2 [file 41419_2023_5827_MOESM3_ESM.tif]

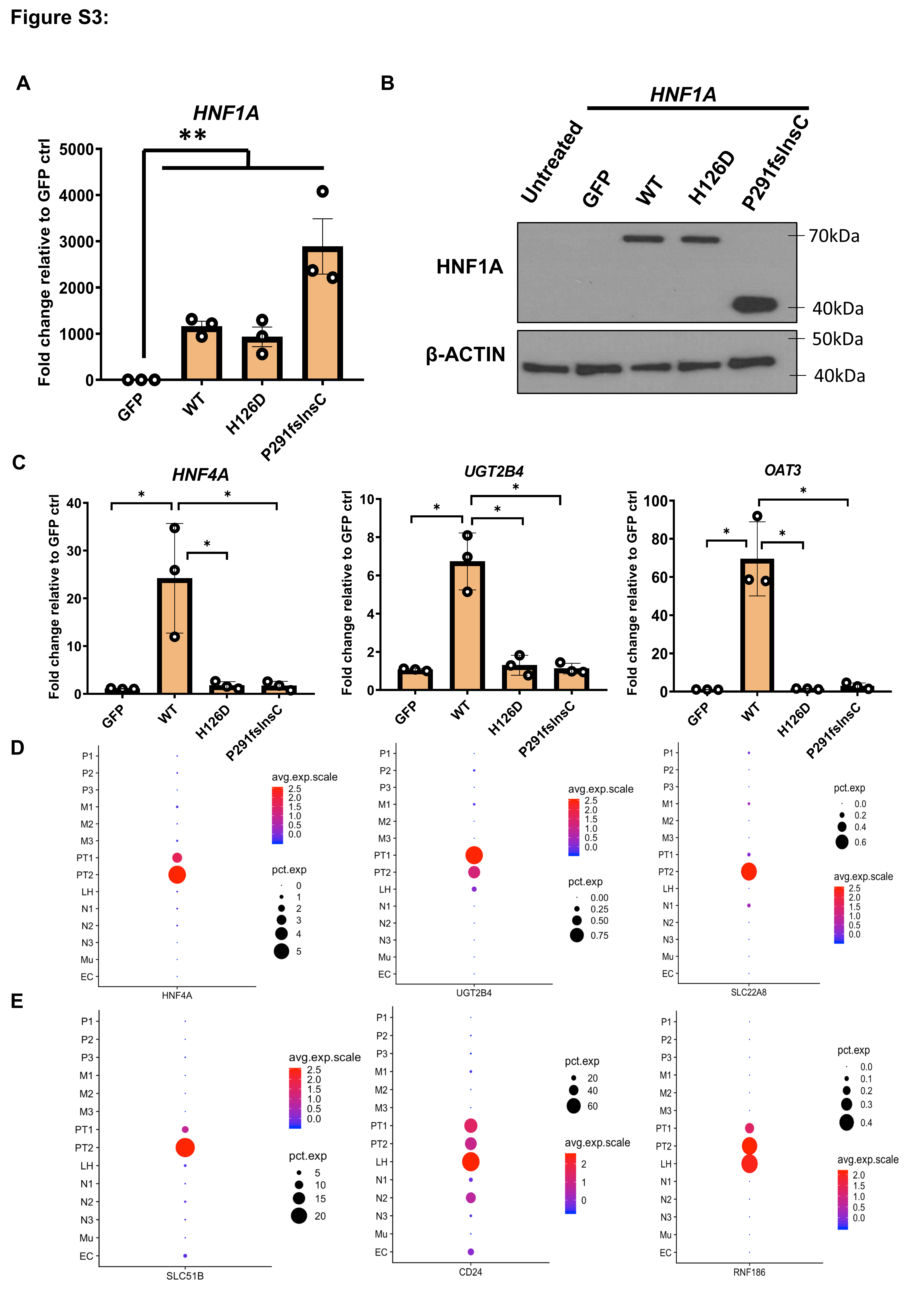

Supplement: Supplementary file 4 — Figure S3 [file 41419_2023_5827_MOESM4_ESM.tif]

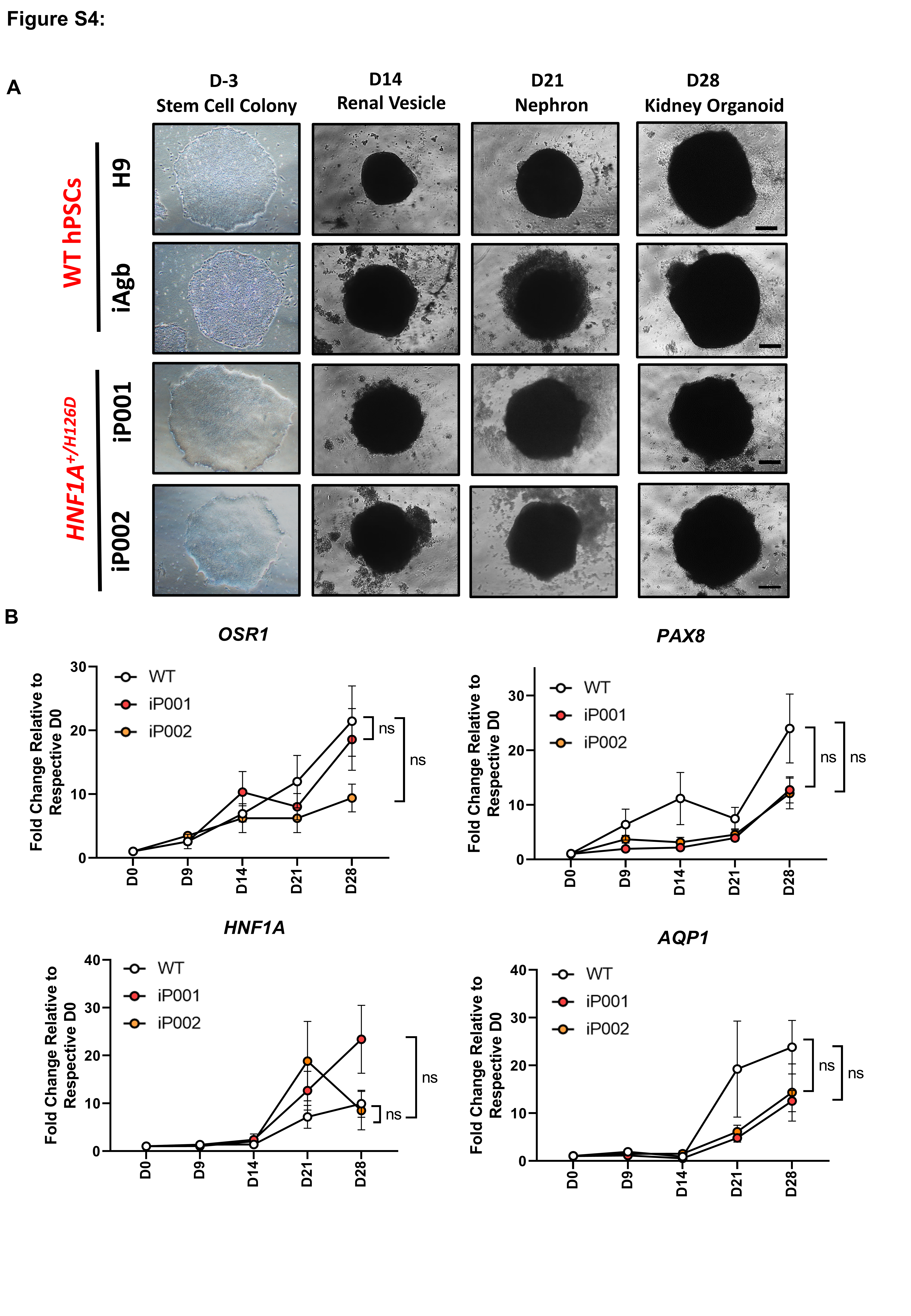

Supplement: Supplementary file 5 — Figure S4 [file 41419_2023_5827_MOESM5_ESM.tif]
