# Supplementary material for: HNF1A binds and regulates the expression of SLC51B to facilitate the uptake of estrone sulfate in human renal proximal tubule epithelial cells
Source: Cell Death Dis. 2023 May 3;14(5):302. doi: 10.1038/s41419-023-05827-8 (PMC10156747; doi:10.1038/s41419-023-05827-8)
Supplement: Supplementary file 12 — Raw WB Images [file 41419_2023_5827_MOESM12_ESM.pptx]

## Slide 1
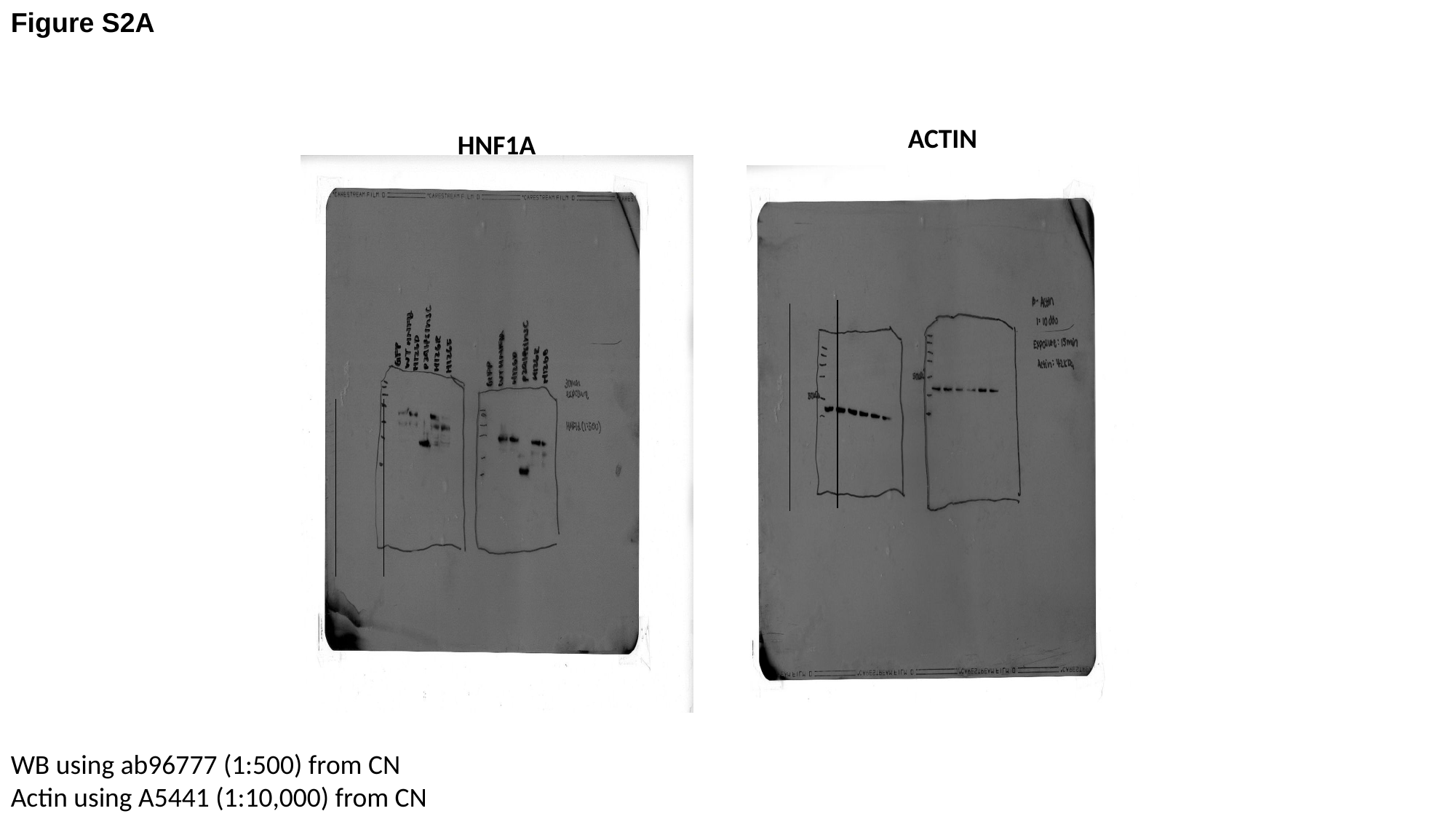

Figure S2A
ACTIN
HNF1A
WB using ab96777 (1:500) from CN
Actin using A5441 (1:10,000) from CN

## Slide 2
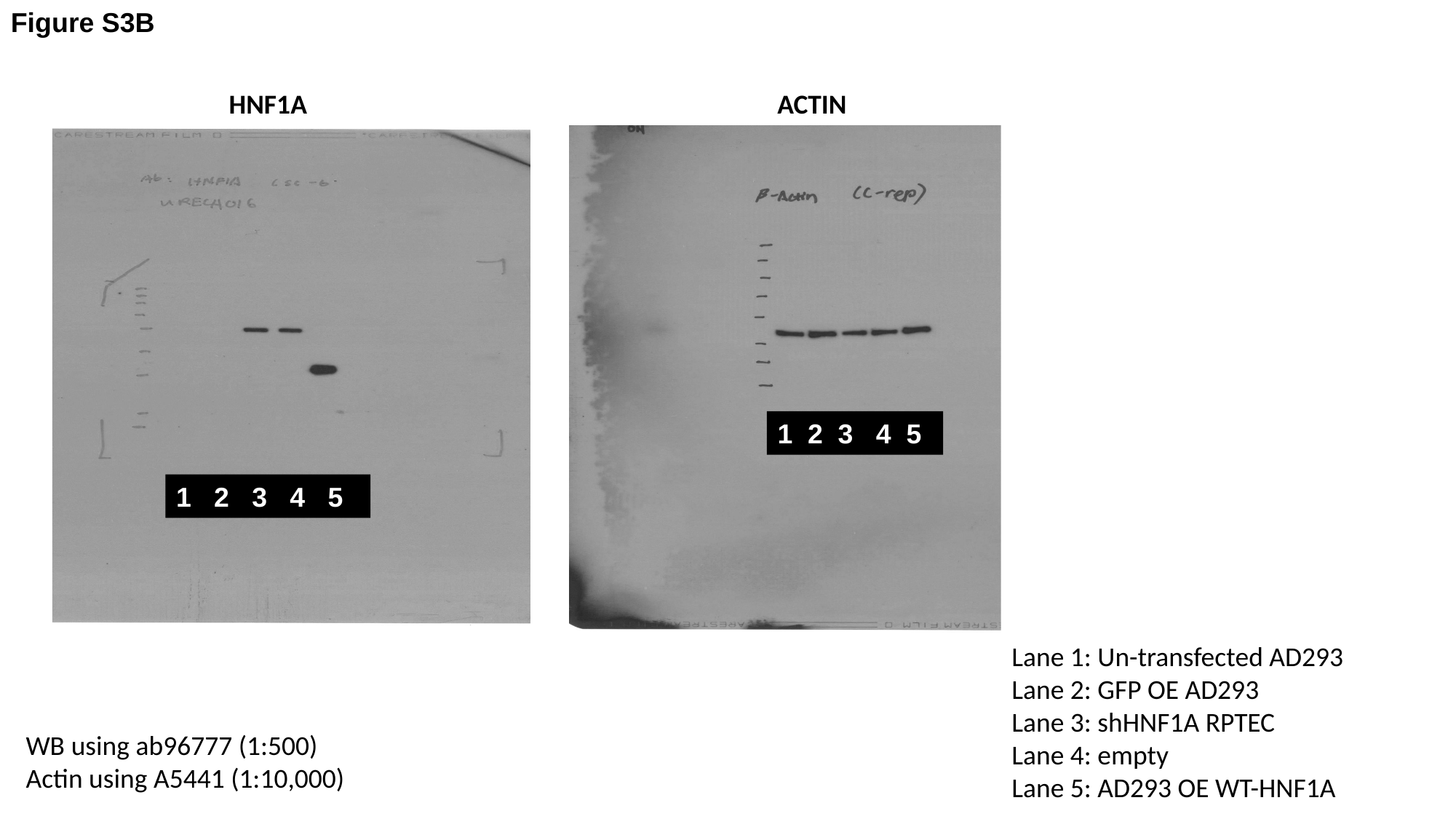

Figure S3B
HNF1A
ACTIN
1 2 3 4 5
1 2 3 4 5
Lane 1: Un-transfected AD293
Lane 2: GFP OE AD293
Lane 3: shHNF1A RPTEC
Lane 4: empty
Lane 5: AD293 OE WT-HNF1A
WB using ab96777 (1:500)
Actin using A5441 (1:10,000)

## Slide 3
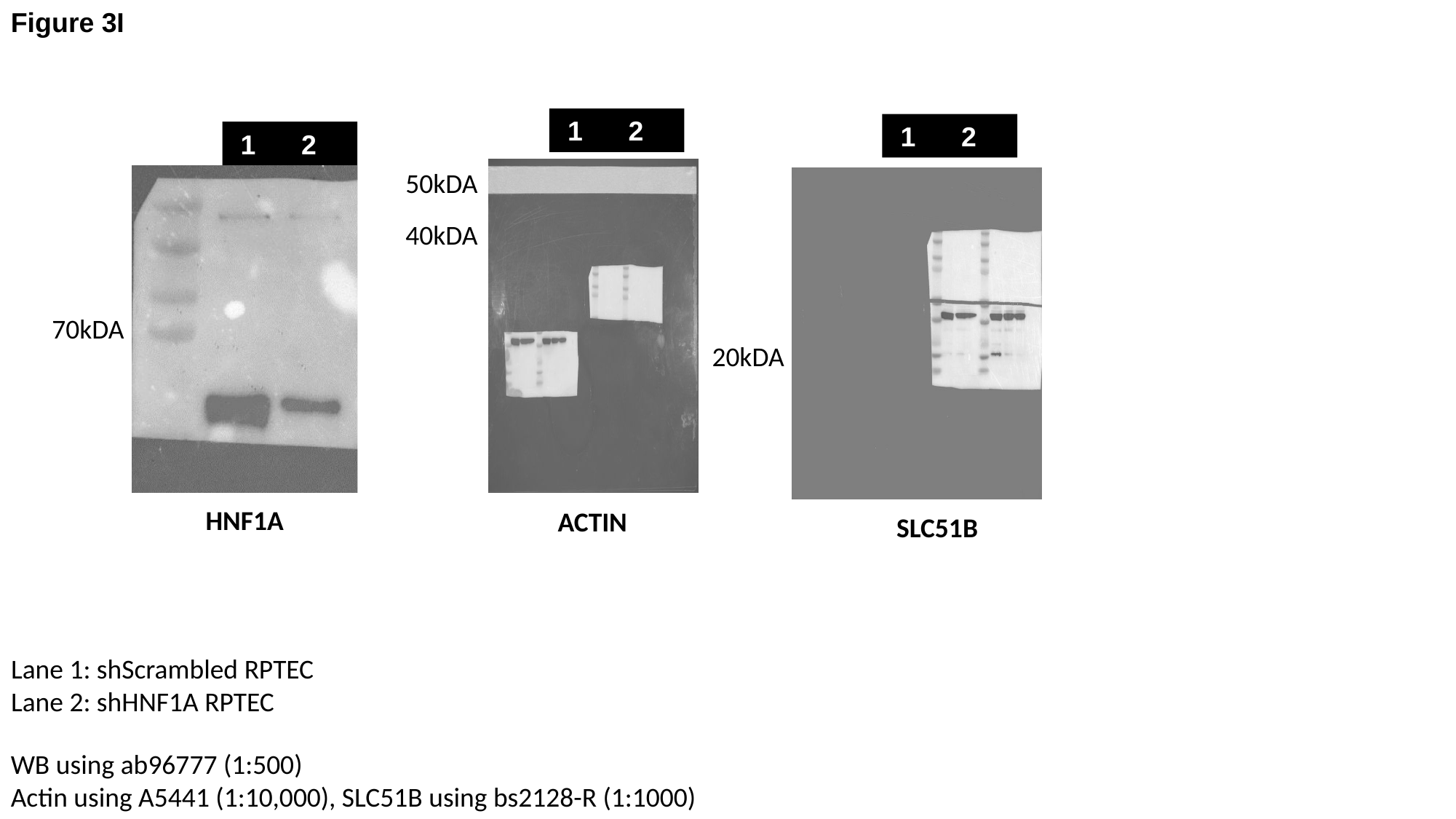

Figure 3I
 1 2
 1 2
 1 2
50kDA
40kDA
70kDA
20kDA
HNF1A
ACTIN
SLC51B
Lane 1: shScrambled RPTEC
Lane 2: shHNF1A RPTEC
WB using ab96777 (1:500)
Actin using A5441 (1:10,000), SLC51B using bs2128-R (1:1000)
