# Supplementary material for: HNF1A binds and regulates the expression of SLC51B to facilitate the uptake of estrone sulfate in human renal proximal tubule epithelial cells
Source: Cell Death Dis. 2023 May 3;14(5):302. doi: 10.1038/s41419-023-05827-8 (PMC10156747; doi:10.1038/s41419-023-05827-8)
Supplement: Supplementary file 13 — Ethics Approval Letter [file 41419_2023_5827_MOESM13_ESM.pdf]

National Healthcare Group
Adding years of healthy life

3 Fusionopolis Link
#03-08 Nexus@one-north
Singapore 138543
Tel: 6496 6600 Fax:6486 6870
www.nhg.com.sg
RCB No. 200002150H

NHG DSRB Ref: **2013/01068**

14 November 2018

A/Prof Lim Su Chi
Department of Diabetics
Khoo Teck Puat Hospital

Dear A/Prof Lim

RENEWAL OF NHG DOMAIN SPECIFIC REVIEW BOARD (DSRB) APPROVAL AND EXEMPTION FROM CONTINUING REVIEW

STUDY TITLE: Generation of human induced pluripotent stem cells (hiPSCs) from subjects with a novel mutation in HNF1A gene for in vitro disease modelling of maturity onset diabetes of the young 3 (MODY3).

We are pleased to inform you that the NHG DSRB has renewed the approval for the application as titled above, being conducted in **Khoo Teck Puat Hospital.** Your study shall be exempted from continuing review as long as the study activities are limited to data analysis only.

The documents reviewed are:

a) NHG DSRB Study Status Report Form ID: **2013/01068-SRF0005**
b) NHG DSRB Application Form: **Version No. 3**
c) KTPH Data Collection Form: **Version 1 dated 01 April 2014**
d) Participant Information Sheet & Informed Consent Form: **Version 2 dated 08 September 2014**

Continued approval is conditional upon your compliance with the following requirements:

1. No deviation from, or changes of the protocol should be implemented without documented approval from the NHG DSRB, except where necessary to eliminate apparent immediate hazard(s) to the study subjects.

2. Any deviation from, or a change of, the protocol to eliminate an immediate hazard should be promptly reported to the NHG DSRB within seven calendar days.

3. If the amendments have changed the study status such that it no longer involves data analysis only, then continuing review shall be required. The new study status must be reported using the NHG DSRB Study Status Report Form immediately.

4. Please note that for studies requiring CTA/CTN/CTC, apart from the approval from NHG DSRB, no deviation from, or changes of the Research Protocol and Informed Consent Form should be implemented without documented approval from the Health Sciences Authority unless otherwise advised by the Health Sciences Authority.

5. Please submit the following to the NHG DSRB:

a. All Unanticipated Problems Involving Risk To Subjects Or Others (UPIRTSOs) must be reported to the NHG DSRB. For more than minimal risk studies, all problems involving local deaths must be reported as soon as possible, but not later than **7 calendar days** after first knowledge by the Investigator, regardless of the causality and expectedness of the death event, and any additional relevant information about the death should be reported within **8 calendar days** of making the initial report. For no more than minimal risk studies, only problems involving local deaths that are related or possibly related to the study must be reported as soon as possible, but not later than **7 calendar days** after first knowledge by the Investigator, and any additional relevant information about the death should be reported within **8 calendar days** of making the initial report. For problems which are life threatening, it should be reported as soon as possible, but not later than **7 calendar days** after first knowledge by the investigator, and any additional relevant information about the problems should be reported within **8 calendar days** of making the initial report. All other problems that fulfil the UPIRTSOs reporting criteria must be reported as soon as possible but not later than **15 calendar days** after first knowledge by the Investigator.

b. Report(s) on any new information that may adversely affect the safety of the subject or the conduct of the study.

c. Study completion – this is to be submitted using the NHG DSRB Study Status Report Form within 4 to 6 weeks of study completion or termination.

Established since May 2006, the NHG Research Quality Management (RQM) Program seeks to promote the responsible conduct of research in a research culture with high ethical standards, identify potential systemic weaknesses and make recommendations for continual improvement. Hence, this research study may be randomly selected for a review by the Research Quality Management (RQM) team. For more information, please visit www.research.nhg.com.sg.

The NHG DSRB operates in accordance to the ICH GCP, and all applicable laws and regulations.

Yours Sincerely

Dr Yong Wei Peng
Chairman
NHG Domain Specific Review Board C

Cc: Institutional Representative, KTPH
c/o Clinical Research Unit, KTPH
Departmental Representative of Diabetics, KTPH

(This is an electronic-generated letter. No signature is required.)
