# Supplementary material for: HNF1A binds and regulates the expression of SLC51B to facilitate the uptake of estrone sulfate in human renal proximal tubule epithelial cells
Source: Cell Death Dis. 2023 May 3;14(5):302. doi: 10.1038/s41419-023-05827-8 (PMC10156747; doi:10.1038/s41419-023-05827-8)
Supplement: Supplementary file 14 — Informed Consent Form [file 41419_2023_5827_MOESM14_ESM.pdf]

INFORMED CONSENT FORM

1. Study Information

Protocol Title:
Generation of human induced pluripotent stem cells (hiPSCs) from subjects with a novel mutation in *HNF1A* gene for *in vitro* disease modelling of maturity onset diabetes of the young 3 (MODY3).

Study Investigators & Contact Details:
A/Prof Lim Su Chi
Senior Consultant
Department of Medicine
Khoo Teck Puat Hosptial
Telephone: 66022353

2. Purpose of the Research Study

You are invited to participate in a research study. It is important to us that you first take time to read through and understand the information provided in this sheet. In addition, before you take part in this research study, the study will be explained to you and you will be given the chance to ask questions. After you are properly satisfied that you understand this study, and that you wish to take part in the study, you must sign this informed consent form. You will be given a copy of this consent form to take home with you.

You are invited to consider this study because you have diabetes resulting from a novel mutation in the *HNF1A* gene. This study is aimed at producing a disease model for the study of diabetes.

This pilot study will recruit 2 subjects (already identified to possess the novel mutation) and 1 or 2 family members (if available) as controls from Alexandra Health-Khoo Teck Puat Hospital. The expected duration of the study period is approximately two years.

3. What procedures will be followed in this study

If you take part in this study, approximately 20 millilitres of blood (about a table-spoon) will be taken from your vein (blood vessel) and a 4 mm skin biopsy will be collected.

We will take a detailed diabetes-related family history from you. The collected blood samples will be used to generate human induced pluripotent stem cells (hiPSCs) for the study of the diabetes disease model. Genetic counseling is available at no charge to all eligible study participants. Hence, you are encouraged to attend the counseling sessions and keep your primary diabetes-care provider informed of your participation in this study.

Your participation in the study will last for about 1 hour.

The DNA obtained from your blood sample may be used for the development of new therapies, diagnostic methods, medicines, treatments for disease, information, materials and other developments which may be patented or otherwise have commercial value.

In addition, your laboratory and relevant clinical data will be obtained either from you or existing medical records. Investigators will place your coded information in a database and will store (“bank”) your blood and skin samples for up to 25 years (under PI’s supervision/ custodian) in freezers located at KTPH, for use in diabetes related research studies in KTPH now and in the future. Your genetic material will not be used for cloning another human being. When (or before) the 25 year period ends, your blood sample will be destroyed. Your health and medical information collected for the study will be retained for up to 25 years. Collected samples and medical information will not be provided to other non-collaborating researchers, institution and commercial biomedical companies.

Your samples will be de-identified in accordance with practice by Proper Conduct of Research. Therefore, samples will not be labeled with information that directly reveal your identity but will be coded with a study-specific identification number. In preparation of the long-term follow-up phase of the study, with appropriate ethical approval, the investigators may link-up future occurrence of any illness related to diabetes and its complication to your baseline data i.e. collected upon entry into the study. Thus, linking-up your de-identified samples with your clinical data is possible. However, this is kept strictly confidential under the joint-custodian of the lead investigators listed at the beginning of this document. The procedure of linking-up will only be performed when appropriate ethical approval is available.

Given the experimental nature of this study, it is in your best interest that the findings will not be communicated to you directly. However, the findings can be disclosed to your regular diabetes-care provider (upon his/her request) only if he/she is a registered-practitioner of the institutions hosting this study.

4. Your Responsibilities in This Study

If you agree to participate in this study, you should follow the advice given to you by the study team. At the present phase of this study, you should be prepared to visit the hospital once and undergo all the procedures that are outlined above.

5. What Is Not Standard Care or Experimental in This Study

The study is being conducted because the contribution of monogenic mutation to diabetes is largely unknown. We hope that your participation will help us better understand the relationship between mutation in this gene and diabetes.

The procedures mentioned above (point 3) are only being performed for the purposes of research and are not part of your routine care.

6. Possible Risks and Side Effects

Obtaining blood can cause pain, bleeding, bruising, or swelling at the site of the needle stick. Fainting sometimes occurs and infection rarely occurs.

Any blood or tissue specimens obtained during the course of this study will be stored and processed for the purposes of this study and other diabetes-related research for a period not exceeding 25 years. The genetic material will not be used for any other purpose.

Genetic disease runs in the family and can be passed-on to future generations. Therefore, we strongly encourage you to attend the relevant genetic counseling session made-available at no charge, if you are an eligible participant of this study. However, as mentioned above, given the experimental nature of this study, it is in your best interest that the findings will not be communicated to you directly. Nevertheless, the findings can be disclosed to your regular diabetes-care provider (upon his/her request) only if he/she is a registered-practitioner of the

institutions hosting this study.

7. Possible Benefits from Participating in the Study

Participation in this study will contribute to the understanding of the genetic basis of their diabetic condition resulting from this novel mutation in *HNF1A* gene. Therefore, establishing the relationship between mutation in this gene and diabetes may guide your diabetes-care provider when considering the choice of therapy for diabetes. Hence, you are advised to consult your regular diabetes-care provider on your diabetes treatment.

There is no other known medical benefit from participation in this study. However, your participation in this study may add to the medical knowledge about the genetic basis of diabetes.

8. Alternatives to Participation

If you choose not to take part in this study, you will continue to receive standard care for your condition, such as medications for diabetes, blood pressure, cholesterol, regular screenings, and consultation with your doctor.

9. Costs & Payments if Participating in the Study

If you take part in this study, the procedures mentioned above (point 3) will be performed at no charge to you.

By consenting to participate in this study, you authorize the use of your sample for the research, and you acknowledge that there are no plans to provide financial benefits or compensation to you should new or patented products, therapies, diagnostic methods, medicines, treatments, information, materials or other developments of commercial value be developed. You will not be reimbursed for your time and transportation costs. However, as a token of appreciation for the inconvenience caused, a small gift or commodities voucher (with a value of $100) will be given to each study participant who has fulfilled the study protocol requirement.

10. Voluntary Participation

Your participation in this study is voluntary. You may stop participating in this study at any time. Your decision not to take part in this study or to stop your participation will not affect your medical care or any benefits to which you are entitled. If you decide to stop taking part in this study, you should inform the lead investigators whose names are listed at the top of this consent form. Upon withdrawal of your consent to participate in this study, all your samples bio-banked in our laboratory will be destroyed.

Your doctor, the Investigator of this study may stop your participation in the study at any time if they decide that it is in your best interests. They may also do this if you do not follow instructions required to complete the study adequately. If you have other medical problems or side effects, the doctor and/or nurse will decide if you may continue in the research study.

In the event of any new information becoming available that may be relevant to your willingness to continue in this study, you will be informed in a timely manner by the lead investigators or his/her representative. The investigators or his/her representative may also contact you in future during the subsequent long-term follow-up phase of the study (targeted at the occurrence of diabetes and related complications) to understand your willingness to participate in the next-phase of this study.

11. Compensation for Injury

If you follow the directions of the doctors in charge of this study and you are physically injured due to the trial procedure properly given under the plan for this study, the Alexandra Health-Khoo Teck Puat Hospital will pay the medical expenses for the treatment of that injury.

Payment for management of the normally expected consequences of your treatment will not be provided by the Alexandra Health-Khoo Teck Puat Hospital. By signing this consent form, you will not waive any of your legal rights or release the parties involved in this study from liability for negligence.

12. Confidentiality of Study and Medical Records

Information collected for this study will be kept confidential. Your records, to the extent of the applicable laws and regulations, will not be made publicly available.

However, the Regulatory Agencies (HSA) and NHG Domain-Specific Review Board and Ministry of Health will be granted direct access to your original medical records to check study procedures and data, without making any of your information public. By signing the Informed Consent Form attached, you are authorizing such access to your study and medical records.

Data collected and entered into the Case Report Forms are the property of Alexandra Health-Khoo Teck Puat Hospital. In the event of any publication regarding this study, your identity will remain confidential.

13. Who To Contact if You Have Questions

If you have questions about this research study, you may contact either the Principal Investigator, A/Prof Lim Su Chi at 66022353.

If you want an independent opinion of your rights as a research subject you may contact the NHG Domain-Specific Review Board Secretariat at 6471-3266.

If you have any complaints about this research study, you may contact the Principal Investigator or the Domain Specific Review Board Secretariat.

CONSENT FORM

Protocol Title:
Generation of human induced pluripotent stem cells (hiPSCs) from subjects with a novel mutation in *HNF1A* gene for *in vitro* disease modelling of maturity onset diabetes of the young 3 (MODY3).

Lead Investigators & Contact Details:
A/Prof Lim Su Chi
Senior Consultant
Department of Medicine
Khoo Teck Puat Hosptial
Telephone: 6602 2353

I voluntarily consent to take part in this research study. I have fully discussed and understood the purpose and procedures of this study. This study has been explained to me in a language that I understand. I have been given enough time to ask any questions that I have about the study, and all my questions have been answered to my satisfaction.

________________________ ______________________________ __________________
Name of Participant Signature Date

☐ Yes, I agree to donate my biological specimen for future research and there are no restrictions on the kind of research that may be done with my biological specimen.

☐ Yes, the investigator may use my biological specimen for future research only when the research is related to diabetes.

☐ No, I do not agree to donate my biological specimen for future research.

Translator Information
The study has been explained to the participant / legally acceptable representative in
__________________________________ by ______________________________________

Witness Statement
I, the undersigned, certify to the best of my knowledge that the participant signing this informed consent form had the study fully explained in a language understood by him / her and clearly understands the nature, risks and benefits of his / her participation in the study.

________________________ ______________________________ __________________
Name of Witness Signature Date

Investigator Statement
I, the undersigned, certify that I explained the study to the participant and to the best of my knowledge the participant signing this informed consent form clearly understands the nature, risks and benefits of her participation in the study.

________________________ ______________________________ __________________
Name of Investigator / Signature Date
Person administering consent
